# Design of high-specificity binders for peptide–MHC-I complexes

**DOI:** 10.1126/science.adv0185

**Published:** 2025-07-24

**Authors:** Bingxu Liu, Nathan F. Greenwood, Julia E. Bonzanini, Amir Motmaen, Jeremy Meyerberg, Tao Dao, Xinyu Xiang, Russell Ault, Jazmin Sharp, Chunyu Wang, Gian Marco Visani, Dionne K. Vafeados, Nicole Roullier, Armita Nourmohammad, David A. Scheinberg, K. Christopher Garcia, David Baker

**Affiliations:** 1 Department of Biochemistry, University of Washington, Seattle, WA, USA.; 2 Institute for Protein Design, University of Washington, Seattle, WA, USA.; 3 Bioengineering Graduate Program, University of Washington, Seattle, WA, USA.; 4 Molecular Pharmacology Program, Memorial Sloan Kettering Cancer Center, New York, NY, USA.; 5 Departments of Molecular and Cellular Physiology and Structural Biology, Stanford University School of Medicine, Stanford, CA, USA.; 6 Department of Pediatrics, Children’s Hospital of Philadelphia, Philadelphia, PA, USA.; 7 Department of Pediatrics, Perelman School of Medicine at the University of Pennsylvania, Philadelphia, PA, USA.; 8 Paul G. Allen School of Computer Science and Engineering, University of Washington, Seattle, WA, USA.; 9 Department of Physics, University of Washington, Seattle, WA, USA.; 10 Department of Applied Mathematics, University of Washington, Seattle, WA, USA.; 11 Fred Hutchinson Cancer Center, Seattle, WA, USA.; 12 Weill Cornell Medicine, New York, NY, USA.; 13 Howard Hughes Medical Institute, University of Washington, Seattle, WA, USA.

## Abstract

Class I major histocompatibility complex (MHC-I) molecules present peptides derived from intracellular antigens on the cell surface for immune surveillance. Proteins that recognize peptide–MHC-I (pMHCI) complexes with specificity for diseased cells could have considerable therapeutic utility. Specificity requires recognition of outward-facing amino acid residues within the disease-associated peptide as well as avoidance of extensive contacts with ubiquitously expressed MHC. We used RFdiffusion to design pMHCI-binding proteins that make extensive contacts with the peptide and identified specific binders for 11 target pMHCs starting from either experimental or predicted pMHCI structures. Upon incorporation into chimeric antigen receptors, designs for eight targets conferred peptide-specific T cell activation. Our approach should have broad utility for both protein- and cell-based pMHCI targeting.

Class I major histocompatibility complex (MHC-I) molecules are cell surface proteins that present peptides derived from intracellular proteins. The recognition of peptides displayed on MHC-I (pMHCI complexes) by the T cell receptor (TCR) enables the immune system to detect and destroy cells expressing proteins associated with disease, as a consequence of infection or mutations. From a therapeutic perspective, targeting pMHCs with engineered cells (1, 2) or proteins—for example, bispecific T cell engagers (BiTEs) (3–5)—is attractive, as it provides an opportunity to distinguish cells on the basis of their intracellular proteins. TCRs can be used for such targeting, but, in many cases, TCRs with specificity to recognize cells expressing disease-associated proteins have not been identified. The diversity of MHC alleles (also referred to as human leukocyte antigen, or HLA, alleles) and antigenic peptides across patient populations necessitates the development of hundreds to thousands of effective and specific binders to achieve broad patient coverage. Current methods rely on empirical TCR screening from patient samples (6) or antibody-like constructs, such as single-chain variable fragment (scFv) libraries (1, 3), which are costly, labor-intensive, and time-consuming. Recently, an engineered system capable of achieving specific MHC-I targeting across different HLA alleles has also been described (40). Efficient methods to generate small, stable proteins that recognize specific pMHCIs of interest could have considerable therapeutic utility for generating recognition domains in chimeric antigen receptors (CARs) for both cell-based and protein-based therapies. To be effective and safe in cell therapy settings, CARs that incorporate designed proteins must mediate specific activation by the target peptide loaded on the target HLA, but not by the thousands of other peptides from the proteome loaded on the same HLA, or by different HLAs—which poses a considerable challenge for binder discovery.

Recent advances in deep learning–driven binder design have enabled the structure-based design of small, stable binders with high affinity and specificity against a wide range of folded (7–9) and disordered (10, 11) protein targets. We reasoned that if such binders could be generated against pMHCIs, there could be multiple advantages in stability, engineerability, and manufacturability, and we set out to explore the computational design of high-affinity and high-specificity pMHCI binders.

## Computational design of pMHCI binders for high target specificity

**Fig. 1.**
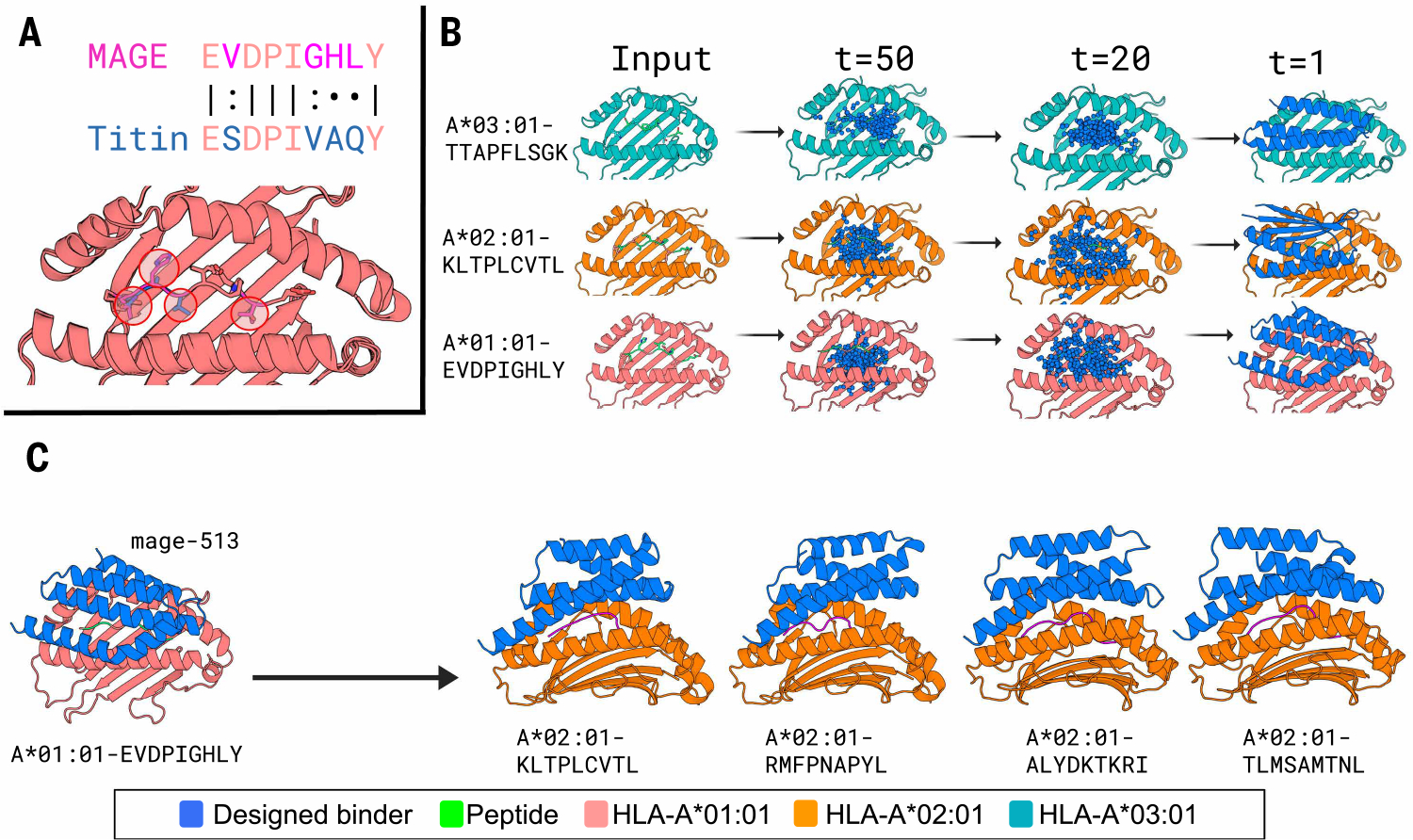
Diffusion of pMHC binders. (**A**) pMHC structure and design challenge. The goal is to distinguish a target peptide (in this example, MAGE) from a closely related off-target (Titin). The positions that differ between the two peptides are circled in the pMHC structure model at the bottom. (**B**) Representative diffusion trajectories and design models for three different pMHC targets. Column 1, target pMHC; column 2, initial Gaussian noise initialization; columns 3 and 4, intermediate steps in diffusion denoising trajectories starting from completely random residue distributions; right column, fully denoised design model backbones. (**C**) Partial diffusion of starting scaffolds against related targets. Single-letter abbreviations for the amino acid residues are as follows: A, Ala; C, Cys; D, Asp; E, Glu; F, Phe; G, Gly; H, His; I, Ile; K, Lys; L, Leu; M, Met; N, Asn; P, Pro; Q, Gln; R, Arg; S, Ser; T, Thr; V, Val; W, Trp; and Y, Tyr.

To functionally substitute for TCRs, pMHC binders must be capable of distinguishing the target peptide from other proteome peptides when presented in the MHC groove (Fig. 1A). To generate such binders, we used the generative AI method RFdiffusion targeted to the outward-facing residues of the bound peptide to generate protein backbones that arc above the central peptide binding groove of MHC-I (Fig. 1B). From many independent trajectories (examples in fig. S1A), we selected backbones capable of hosting side chains that interact extensively with the peptide (12) (fig. S1B), optimized their sequences for folding and binding using ProteinMPNN, and selected those that AlphaFold2 (AF2) (13) predicted to fold and bind as designed. We then used ProteinMPNN to predict the sequence of the target peptide in the context of the designed binder and selected those designs for which the confidence of the on-target peptide sequence was higher than off-target sequences (fig. S1C). Finally, we used a fine-tuned AF2 model (14) to predict the structure of each design with the target peptides and closely related peptides in the proteome in complex with the MHC and selected designs predicted to bind the on-target peptide considerably more confidently than the off-target peptides (fig. S1D).

## Designed binders bind to their target pMHCI with high affinity and specificity

**Fig. 2.**
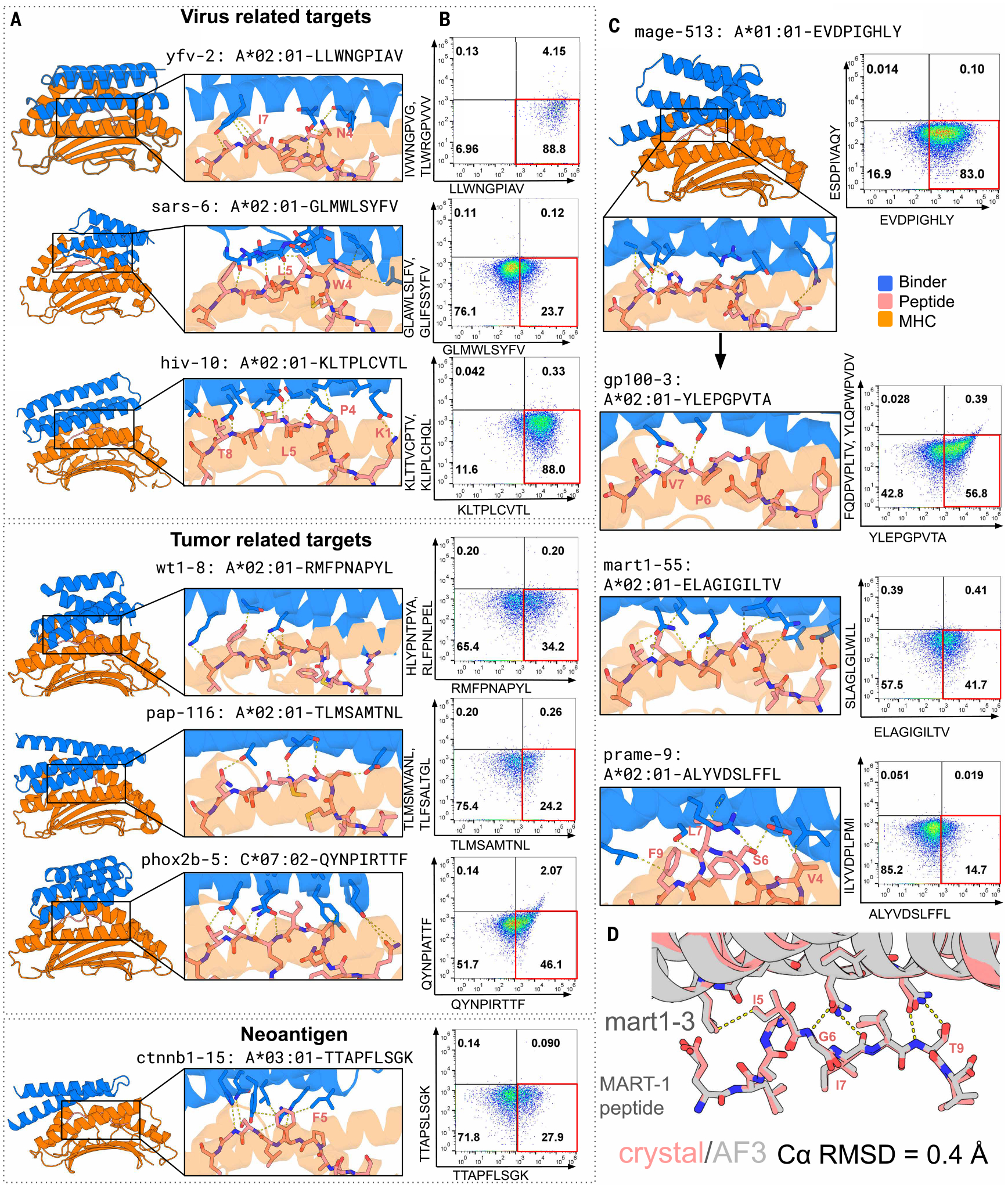
Design models and binding specificity. (**A**) Design models. Left, overall structure; right, zoom in on peptide binding region. pMHCI in orange, peptide in sticks and salmon, and binder in blue. HLA allele and peptide sequence are specified above the zoom-in view. (**B**) Flow cytometry of cells displaying the design incubated with on-target pMHCI tetramer (*x* axis) and one or two off-target tetramers (*y* axis) at 10 nM concentration. Staining in the lower-right quadrant indicates specific on-target binding. Row 1, individual designs displayed on yeast; rows 2 to 7, CARs incorporating designs on Jurkat cells. (**C**) Partial diffusion of design at top to targets below (left panels) and corresponding Jurkat straining (right). (**D**) Crystal structure of mart1–3 binder overlaid with AF3 structure prediction.

To evaluate the ability of our design pipeline to generate binders to a wide range of pMHCI complexes, we selected a structurally diverse set of 11 complexes comprising HLA alleles A*01:01, A*02:01, A*03:01, and C*07:02 presenting 9- and 10-mer peptides from viral proteins, tumor-associated proteins, and neoantigens (data S1). For each pMHCI target, we obtained oligonucleotide pools encoding between 200 and 12,000 designs, displayed them on yeast, and selected those that specifically recognized the targeted pMHCI but not one to four closely related off-target peptides (table S1) on the same MHC using FACS (fluorescence-activated cell sorting) to collect cells displaying designs that bound on-target (labeled with one fluorescent marker) but not off-target (labeled with a different color marker) pMHCI tetramers (fig. S2A, lower-right quadrant) followed by next-generation sequencing enrichment analysis or clonal selection to determine their identities (Fig. 2A).

For eight of the pMHCI complexes, we used the de novo diffusion approach to generate designs with a range of topologies (Fig. 2A, left) and peptide binding interfaces (Fig. 2A, right) that bound the target peptide specifically, with reduced binding to off-target peptides on the same HLA (Fig. 2B). Predictions of the structures of the designed proteins in complex with their cognate pMHCI using AlphaFold3 (AF3) and Chai-1 (15, 16) were very similar to the design models (table S2 and fig. S2, B and C, right).

To evaluate the accuracy of our design approach, we obtained a 2.2-Å crystal structure of binder mart1–3 with the MART-1 pMHCI complex. The crystal structure closely matched the design model at both the backbone [C-alpha root mean square deviation (CαRMSD) = 0.4 Å] and side-chain level (interface all-atom RMSD = 0.4 Å across 15 interface residues), with the designed binder forming an extensive interface with the peptide (Fig. 2D, table S3, and fig. S3). Designed interactions between mart1–3 (design naming scheme is “lowercase target name– design number”) and the MART-1 peptide recapitulated in the crystal structure include hydrophobic interactions with the L5 side chain and hydrogen bonds with the main chains of G6, I7, and T9 and the side chain of T9 (Fig. 2D).

Three of the pMHCI targets are viral peptides presented on A*02:01: SARS-CoV1/GLMWLSYFV (17), YFV/LLWNGPIAV (18), and HIV/KLTPLCVTL (19). To assess whether the designs could function when incorporated into CARs, we generated between 10 and 30 CAR constructs per design (20) and expressed them on the surface of Jurkat cells. For each of the three targets, we identified designs that distinguished the target peptides from closely related off-target peptides when displayed on yeast (Fig. 2B, top row) or when incorporated into CARs (Fig. 2B, rows 2 and 3). In the design models (Fig. 2A), these binders make extensive contacts with the target peptide (table S2). Design sars-6 buried the target peptide W4 and L5 in a hydrophobic pocket and made a network of flanking hydrogen bonds with the peptide backbone. Design yfv-2 made hydrogen bonds with N4 and hydrophobic interactions with I7 of the peptide. Design hiv-10 made hydrogen bonds with K1, T8, and the peptide backbone.

Four of the pMHCI design targets were tumor-associated antigens: WT1 (RMFPNAPYL/A*02:01) (21–23), PAP (TLMSAMTNL/A*02:01) (24), PHOX2B (QYNPIRTTF/C*07:02) (1), and the neoantigen CTNNb1 (TTAPFLSGK/A*03:01 with S5F mutation) (25). We identified specific designs for all four peptides (Fig. 2B), which interact extensively with the peptide targets (Fig. 2A). Design ctnnb1–15 makes extensive contact with the mutated F5 through cation-pi, pi-pi, and other hydrophobic interactions (Fig. 2A), likely contributing to specific binding for the S5F peptide versus the unmutated CTNNb1 peptide (Fig. 2B). Design phox2b-5, generated against a predicted pMHCI structure, makes hydrogen bonds to the peptide backbone and side chains, including bidentate interactions to R6 of the peptide backbone, rigidifying the peptide and contributing to the observed specificity against the R6A mutant (Fig. 2A).

## Privileged scaffolds can be redesigned to target multiple pMHCs

While the de novo RFdiffusion approach can generate binders to pMHCI targets, it is computationally expensive, as only a small fraction of trajectories yield backbones that interact extensively with the peptide while largely avoiding the MHC. Given the overall similarity of pMHCI structures, we reasoned that designed backbones that effectively target one pMHCI complex could likely be repurposed to recognize other pMHCI targets. We used partial diffusion (26) to adapt the most promising scaffolds to additional pMHCI targets [Fig. 1C and data S1; partial diffusion carries out partial noising-denoising to sample around an input structure (7)]. We used partial diffusion starting from a designed binder against ALHGGWTTK/A*03:01 to generate a design (mage-513) that bound the melanoma-associated antigen 3 (MAGE-A3) peptide (EVDPIGHLY/A*01:01) (27, 28) but not the off-target peptide (ESDPIVAQY/A*01:01) (29) derived from the cardiac Titin protein (Fig. 2C, top). From this scaffold, we used partial diffusion to design binders for other 9- and 10-residue tumor-associated antigens presented by A*02:01: the gp100 peptide YLEPGPVTA (30), the MART-1 peptide (A2L) ELAGIGILTV (31), and PRAME (ALYVDSLFFL) (32). For gp100, MART-1, and PRAME, we identified specific binders from partial diffusion that made extensive and diverse hydrophobic and hydrogen bonding interactions with the target peptide (Fig. 2C).

To evaluate the behavior of our designs as soluble proteins, we expressed them in *Escherichia coli* and purified the hiv-10 and mage-513 binder designs. The purified designs eluted as single peaks in size exclusion chromatography around the expected size (fig. S2, B and C, left). Surface plasmon resonance (SPR) showed that they bound their cognate pMHCIs with binding affinities in the single-to double-digit nanomolar range (fig. S2, B and C, middle).

## Designed binders have high specificity

We evaluated the ability of the designs to confer specific T cell activation by the target pMHCI by incorporating them into CARs and expressing them on Jurkat cells. These were incubated with 293T cells treated with on- or off-target peptides. 293T cells naturally express HLA-A*02:01 and C*07:02 and have an intact antigen presentation system, and hence present a wide range of peptides derived from self-proteins on their surfaces (represented as the DMSO condition in all Jurkat activation assays); requiring selective activation (measured by CD69 expression level) by pulsing peptides in this system is thus more stringent than using cells that cannot present self-peptides owing to defects in presentation.

**Fig. 3.**
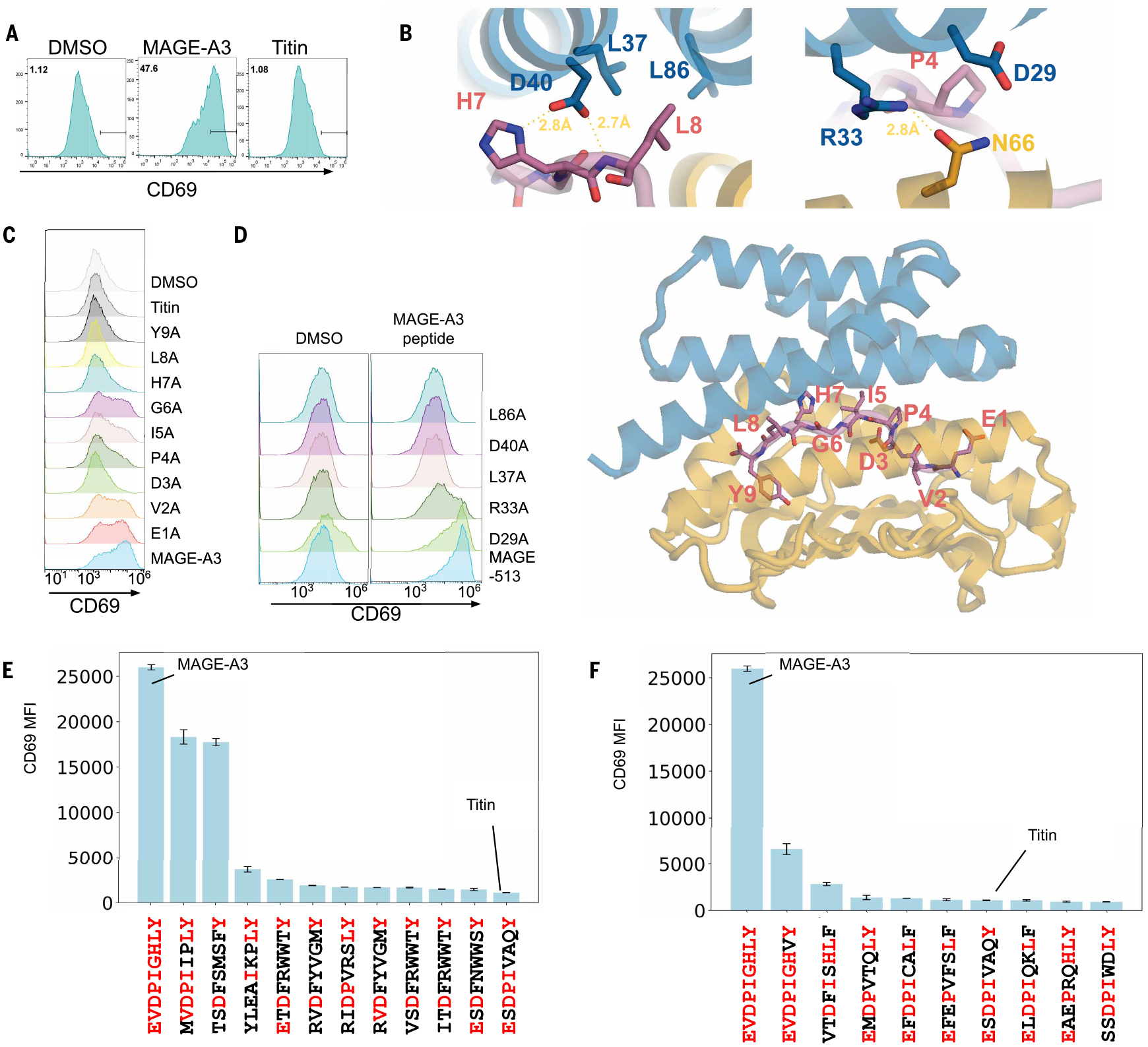
Selective activation of T cells expressing designed CARs targeting MAGE-A3. Activation of Jurkat cells expressing the mage-513 CAR by 293T cells expressing HLA-A*01:01 pulsed with 5 μM of different peptides measured through CD69 expression level. (**A**) Histograms of CD69 expression levels after treatment with 5 μM MAGE-A3, the closely related Titin peptide, or DMSO; the MAGE peptide leads to considerable activation, whereas the Titin peptide is similar to the DMSO control. Horizontal black bars represent CD69 positive population; the fraction of cells within this range is indicated at top left of each panel. (**B**) Design models of the mage-513/MAGE-A3 peptide/HLA-A*01:01 complex (bottom panel) and zoom-in view of the key residues mediating the interaction (top panels). (**C**) Histograms of CD69 surface expression levels by mage-513 CARs after pulsing 293T cells with MAGE-A3 single alanine mutants (D3A indicates mutation of the Asp at position 3 in the peptide to alanine) or DMSO. (**D**) Histograms of CD69 expression levels after pulsing mage-513 variant CARs with MAGE-A3 peptide or DMSO. (**E** and **F**) CD69 levels of Jurkat cells expressing mage-513 CAR upon pulsing with top-ranked peptides from yeast binding screen (E) or from sequence similarity search (F). Residues that are identical to MAGE-A3 peptide at given positions are indicated in red. Experiments were done in duplicate. Error bars indicate standard deviations. MFI, mean fluorescence intensity.

Jurkat cells expressing CARs incorporating the mage-513 design were strongly and specifically activated by MAGE-A3 peptide stimulation but not by the off-target Titin peptide or peptides derived from the intracellular proteome on HLA-A *01:01 (Fig. 3A). Although they bound to the target tetramer specifically (fig. S4A), other designs showed weak (fig. S4B, mage-282) or background activation (fig. S4B, mage-4), likely due to responses to other peptides from the proteome loaded on the same HLA [only HLA-A *01:01–expressing 293T cells induced background activation (fig. S4B)].

In the design model (Fig. 3B), mage-513 engages the MAGE peptide through hydrogen bonds with the side chain of H7 and backbone of L8 (Fig. 3B) and through hydrophobic interactions (mediated by design L37 and L86) with the side chain of peptide L8 (there were also hydrogen bonds between R33 of the design and N66 of the HLA) (Fig. 3B). D29 of the design is close to P4 of the peptide without making direct contact (Fig. 3B); we concluded that this residue likely played a gatekeeper role by clashing with off-target peptides with bulky residues at site 4. Consistent with the design model and predicted structure, mutating residues in the peptide that make extensive interactions to the binder (I5, H7, L8) to alanine disrupted TCR activation (Fig. 3C). CARs with alanine mutations in designed binder residues (L37, D40, L86), which interact most closely with the peptide, showed reduced activation compared with WT mage-513 upon stimulation (Fig. 3D). Mutation of D29 did not influence Jurkat activation upon pulsing with MAGE peptide but did have higher background activation when coincubated with HLA-A*01:01–expressing 293T cells, likely because of increased cross-reactivity, consistent with a gatekeeper role (Fig. 3D).

To further characterize the specificity of mage-513, we used it to probe a comprehensive library of covalently linked peptide-HLA-A*01:01 complexes displayed on yeast (33). The top-hit peptides (data S2) were further tested by target cell pulsing and coincubation with mage-513 CAR–expressing Jurkat cells, and three activating peptides were identified (peptides that bind on yeast but do not activate could be false positives arising from the covalent linkage in the yeast construct). Two of the three had outward-facing side chains similar to those of the MAGE-A3 peptide (Fig. 3E, shown in red). Therefore, we carried out an in silico scan of cross-activating peptides in the human proteome based on sequence similarity (34) and tested these for mage-513 CAR activation. The most activating peptides (EVDPIGHVY and VTDFISHLF) were among the most similar, sharing outward-facing residues I5, H7, and L8 (Fig. 3F), consistent with the alanine scanning results.

**Fig. 4.**
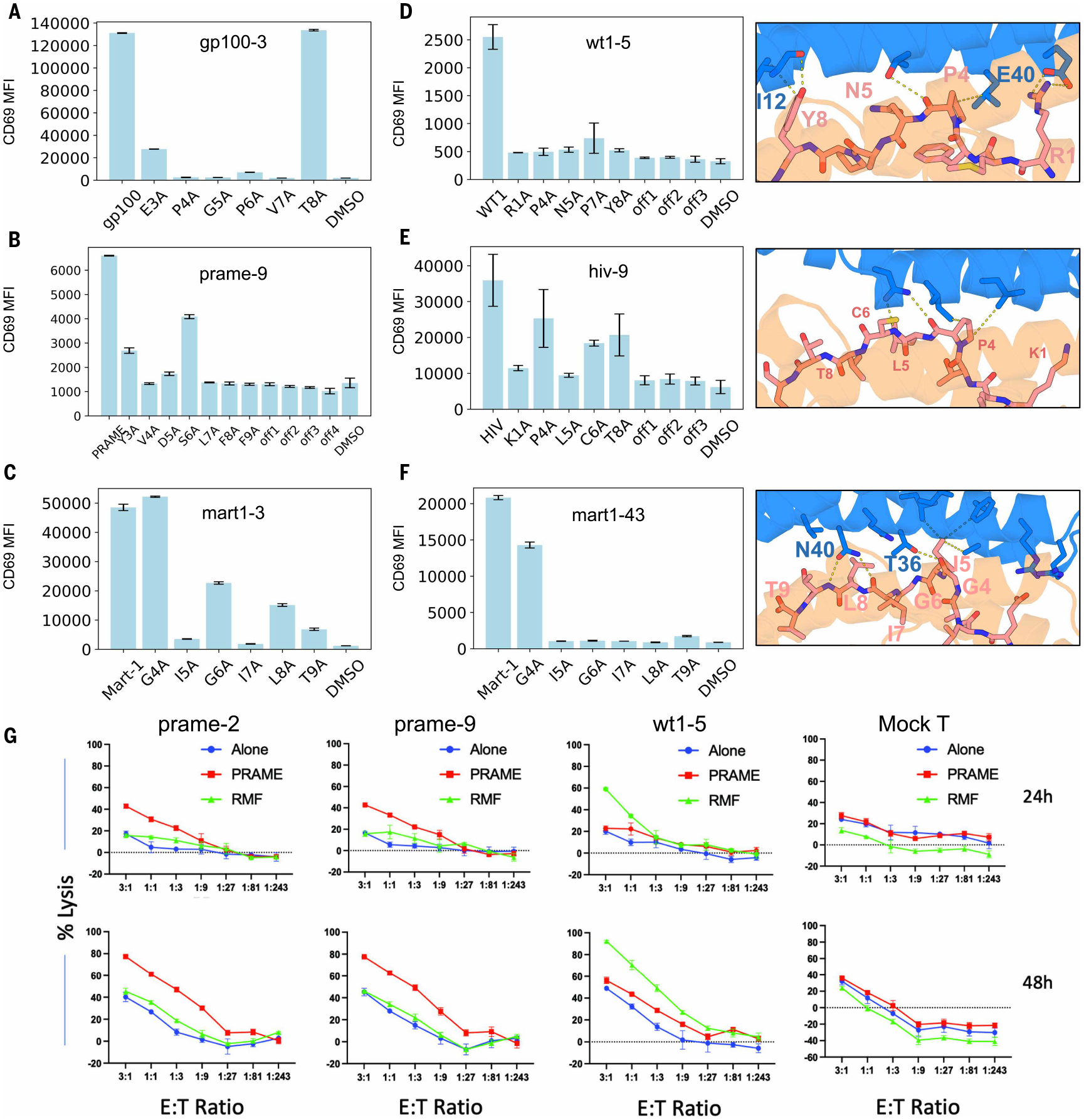
Specific activation and cell killing by designed CARs in T cells through cognate pMHC complexes. Activation of Jurkat cells expressing the CARs by 293T cells with 5 μM of different peptides measured through CD69 expression level by staining (indicated by histogram or MFI). (**A** to **C**) CD69 MFI of (A) gp100–3, (B) prame-9, and (C) mart1–3 CARs with cognate peptide, alanine mutant peptides, or DMSO. (**D** to **F**) (Left) CD69 MFI of (D) wt1–5, (E) hiv-9, and (F) mart1–43. (Right) Zoom-in view of design model with pMHC target. Experiments done in duplicate. Error bars indicate standard deviations. (**G**) CD3 T cells transduced with indicated binder constructs were incubated with HLA-A*02:01+ T2 cells expressing firefly luciferase and green fluorescent protein and pre-pulsed with either the WT1 peptide (RMF) or PRAME at 10 μg/ml overnight at the indicated E:T ratios. Cytotoxicity was measured for each transduced cell (labeled above graph) by bioluminescence emission after adding substrate luciferin, after 24 or 48 hours of incubation. Green, RMF peptide (RMFPNAPYL) pulsed cells; red, PRAME peptide (ALYVDSLFFL) pulsed cells; blue, unpulsed cells. Each experiment was conducted twice with different human donors. One experiment is shown. Each data point and error bar are the mean ± SEM of triplicate measurements.

CARs incorporating binders against the gp100, MART-1, WT1, SARS, and HIV antigens likewise conferred target peptide –selective activation of CD69 signaling in Jurkat cells (Fig. 4, A to F, and fig. S5A). We further probed recognition specificity with peptide alanine scanning experiments. For most targets, pulsing 293T cells with cognate peptide activated at the same level as or at higher levels than pulsing with alanine peptide variants, and mutational effects were consistent with the design models. For gp100–3, substitution of residues P4, G5, P6, and V7 substantially reduced activation; in the design model of gp100–3 (Fig. 2C), there are multiple hydrogen bonds with the backbone of P6 and V7, which likely require precise positioning, making activation sensitive to mutations at the adjacent P4 and G5 (Fig. 4A). For wt1–5, E40 of the binder makes bidentate interactions with peptide residue R1, and the main chain of binder residue I12 makes a hydrogen bond with the side chain of peptide residue Y8, consistent with the alanine scanning results (Fig. 4D). For the MART-1 target, we performed alanine scanning experiments for two different binders, mart1–3 (Figs. 2D and 4C) and mart1–43 (Fig. 4F), which have binding docks centered over different regions of the peptide; this structural shift is reflected in the alanine scanning results observed. For hiv-9, the design makes hydrogen bonds with the peptide backbone and hydrophobic interactions with P4 and L5, likely holding the peptide in place and leading to higher specificity (Fig. 4E).

## Binders against pMHCI targets without experimental structures have high specificity and mediate cell killing

As only a small fraction of potential pMHCI complexes have experimentally determined structures, we also explored the possibility of using predicted pMHCI structures as targets for binder design. The PRAME protein is highly expressed in multiple types of tumors, and a peptide derived from PRAME (ALYVDSLFFL) (32) is displayed on HLA-A*02:01. Despite the strong therapeutic relevance and wide patient population coverage, no high-resolution structure has been determined.

For the design, we experimented with using AF3-predicted pMHCI structures as starting points and identified two binders (prame-9 and prame-2) that specifically induced Jurkat activation upon incubation with 293T antigen-presenting cells and PRAME peptide pulsing (Fig. 4B and fig. S5A). In SPR experiments, the purified designs had dissociation constants for the target pMHCI of 96 and 35 nM, respectively (fig. S5B). Pulsing 293T cells with the original PRAME peptide and single alanine mutant version showed that binder prame-9 was quite specific (Fig. 4B). In the design model (Fig. 2C), the binder interacted with the side chains of V4, S6, L7, and F9 and the main chain of L7, consistent with the observed specificity. To test the generalizability of designing against predicted pMHCIs, we also tested CARs against PHOX2B and identified multiple binders, including phox2b-11 (fig. S5A), that enable specific activation upon incubation with 293T incubating with PHOX2B peptide.

To determine whether specific activation can be converted to specific killing, we transduced primary human T cells with CARs incorporating the designed binders against PRAME and WT1 described above. The designed binder CARs induced killing of HLA-A*02:01+ target T2 cells pulsed with cognate target peptide with higher killing efficiencies than noncognate peptide or nonpulsing controls (Fig. 4G), suggesting that designed binders can mediate specific killing.

## Discussion

A central challenge in targeting pMHCI complexes is achieving high specificity. Screening TCRs from human T cells takes advantage of the natural repertoire but is limited by availability of donors with relevant HLA alleles, central tolerance that can eliminate TCRs most activated by endogenous targets, and the overall rarity of high-affinity TCRs (1, 6). Engineering existing low-affinity TCRs can increase affinity but requires extra effort to ensure specificity to avoid toxicity (29). Screening binders from scFv libraries has primarily yielded nonspecific binders, with specific scFvs adopting similar docking geometry as TCRs (1, 3), suggesting the importance of the docking interface for sufficient peptide contact and binding specificity. These methods have identified specific binders against multiple pMHCIs, but, given the high cost and low hit rate, they are difficult to employ against the large number of potential pMHCI therapeutic targets. Our de novo design approach builds on lessons learned from the binding modes of TCRs and successful scFvs by generating binders with pMHCI interaction interfaces focused on the presented peptide with limited contact with the MHC. We demonstrate that this enables robust design of specific binders for 11 diverse pMHCI targets; because of the simplicity of the structures (compared with scFvs and TCRs), further optimization can be carried out on the basis of the design models and predicted structures without experimental structure determination. Our design pipeline is readily applicable to a wide range of pMHCI targets, allowing for the generation of specific experimentally validated binders within weeks.

We expect the power of our approach to rapidly design specific binders to pMHCIs to continue to increase. First, it should be possible to learn from design campaigns the properties most correlated with specific cell activation and what scaffold geometries give the most effective readouts of sequence over the full peptide length. Second, as deep learning–based structure prediction, design, and model ranking methods improve, it should become possible to find designs with suitable affinities and specificities in testing dozens of candidates. With such advances, it should become possible to readily generate clinical-grade pMHCI binders to provide therapeutic benefits to broad patient populations.

## Supplementary Material

supplementary figures and tables

Data S2

Data S1

MDAR Reproducibility Checklist

science.org/doi/10.1126/science.adv0185

Materials and Methods; Figs. S1 to S5; Tables S1 to S3; References (36–39);

MDAR Reproducibility Checklist; Data S1 and S2

## Data Availability

All data needed to evaluate the conclusions in the paper are present in the paper or the supplementary materials. The mart-1 binder-antigen complex coordinates and structure factors are available in the Protein Data Bank under PDB ID 9O5S/pdb_00009o5s. Code and examples for the computational design of pMHC-I binders are available in Zenodo (35).
