# Supplementary material for: Design of high-specificity binders for peptide–MHC-I complexes
Source: Science. Author manuscript; Available in PMC 2026 Apr 14. (PMC13077772; doi:10.1126/science.adv0185)
Supplement: supplementary figures and tables [file NIHMS2142464-supplement-supplementary_figures_and_tables.pdf]

Supplementary Materials for

Design of high-specificity binders for peptide–MHC-I complexes

Bingxu Liu *et al*.

Corresponding authors: Bingxu Liu, bxliu@uw.edu; David Baker, dabaker@uw.edu

Science **389**, 386 (2025)
DOI: 10.1126/science.adv0185

The PDF file includes:

Materials and Methods
Figs. S1 to S5
Tables S1 to S3
References

Other Supplementary Material for this manuscript includes the following:

MDAR Reproducibility Checklist
Data S1 and S2

Materials and Methods

Computational design of pMHC binders

Target structures used as inputs for binder design were obtained from the Protein Data Bank for A*01:01 MAGE-A3 (PDB: 5BRZ), A*03:01 CTNNb1 (PDB: 6O9C), A*02:01 HIV-Env (PDB: 2X4O, AF3 models), A*02:01 Wilms tumor antigen 1 (PDB: 6RSY, AF3 models), A*02:01 SARS-CoV membrane protein (PDB: 3I6G), A*02:01 YFV NS4b214-22 (PDB: 6SS8), A*02:01 MART-1 (PDB: 5NHT), A*02:01 gp100 (PDB: 5EU3). The structure for A*02:01 PAP (TLMSAMTNL) was predicted by folding with a version of AlphaFold2 fine-tuned for MHC (*14*) structures; the structure for A*02:01 PRAME (ALYVDSLFFL) was predicted by folding with AlphaFold3 using all 5 model outputs. Upward-facing residues of the peptide were chosen as hotspots to condition RFdiffusion towards generating binders with high peptide contact. For scaffold recycling, a partial_t between 12 and 25 (out of 50 total RFdiffusion denoising steps) was used after docking scaffolds to new targets. ProteinMPNN was used to generate sequences for the output backbones. A range of 1,000-20,000 backbones were generated per RFdiffusion cycle, with anywhere between 4-32 MPNN sequences generated per backbone. Output structures were subject to *in silico* screening based on AlphaFold2 initial guess (pAE interaction, binder pLDDT and binder RMSD), AlphaFold2 monomer pLDDT, Rosetta contact molecular surface (CMS) per target residue or range of residues, and ProteinMPNN log probability scores of designs in complex with alanine scan mutants of the target peptide. The cutoff values varied for each target protein and round of design. As a general practice, AF2 pAE_interaction score <5 is the general filter. CMS, ProteinMPNN specificity score, AF2 specificity score were used to rank the preference for ordering among designs. This design process was iterated for each target through new rounds of partial RFdiffusion until a desired number of designs passed the cutoffs.

AF-MHC screening

Building off previous work in predicting pMHC complexes, prior to the release of AF3, we altered the AlphaFold2 fine-tuned for MHC (*14*) to predict minibinder-pMHC complexes through additional templating of the minibinder. For screening, designs were predicted against the on-target and 2-3 relevant off-target peptides in the same HLA allele. The pAE interaction between only the peptide and the minibinder was calculated, and for each off-target a delta pAE interaction (on-target pAE - off-target pAE, more negative is better) was used to filter designs, with scores ranging from -10 to -0.5 depending on the on-target/off-target pair.

Rosetta CMS screening

Rosetta contact molecular surface described before (*12*) uses a triangulation algorithm to calculate the contact surfaces of binder and target and gives it a score taking into account cavities and holes in the interface. For peptide contact screening, we restricted the target surface being calculated by the algorithm to the peptide only or to individual residues of the peptide, and scored its contact to the entire binder surface. Cutoff values were determined empirically for each target.

ProteinMPNN log probability screening

ProteinMPNN was used to score the likelihood of each peptide residue being predicted when in complex with a binder. Binders were screened by scoring each peptide residue separately and filtering directly on the log probability of the on-target residue, or on the difference in log probabilities to when that residue is mutated to an alanine.

Off-target peptide identification

To determine peptides that are likely to cross react with our peptide of interest, we used selective Cross-Reactive Antigen Presentation (*1*), and took all the outputs and cross-checked them with NetMHC4.1 (*36*), using any peptides with an EL_rank <= 0.5. Then we used a simple blossom alignment of the passing peptides against our peptide of interest to rank and choose the top off-target peptides.

DNA library preparation

For yeast surface display, all designed protein sequences were first padded at both termini using GS to a uniform length of either 88 amino acids or 102 amino acids depending on if a 300bp or 350bp oligonucleotide library was ordered, respectively. Then the sequences were reverse translated using dnachisel while codon optimizing for *S. cerevisiae*. DNA sequences were ordered as oligonucleotide libraries via Twist Biosciences or Integrated DNA Technologies (IDT), with 300bp or 350bp sizes.

Yeast surface display screening with FACS

Transformed S. cerevisiae EBY100 strain library cultures were grown in C-Trp-Ura (2% glucose w/v) medium and induced for expression in SGCAA (0.2% glucose w/v) medium. Cells were washed with PBSF (PBS with 1% BSA w/v) and incubated with FITC-conjugated anti-C-Myc chicken antibody (ICL, CMYC-45F) for expression sorting. For binding sorts, cells were additionally incubated with peptide-MHC biotinylated tetramer (Fred Hutchinson Cancer Center Immune Monitoring Services) or dextramer (Immudex) conjugated with phycoerythrin (PE) for on-target peptide or allophycocyanin (APC) for off-target peptide binding. When tetramers or dextramers were mixed, biotin was added to a final concentration of 1uM. Cells were incubated for 30 minutes, then washed and resuspended with PBSF. All sorts were performed on the Sony SH800 FACS instrument. FACS data was analyzed using the FlowJo software. All naive and sorted pools were sequenced using Illumina NextSeq and MiSeq and analyzed for enrichment between sorts.

Protein binder expression and purification

Synthetic genes of top yeast display hits were ordered as eBlock gene fragments from IDT and cloned into a pET29b(+) vector containing an C-terminus His6x tag for all designs and an Avi-tag or SNAC-tag for some designs to introduce Trp to expressed sequences for protein quantification using A280 signal. Following cloning and transformation as in (*37*), we picked single colonies for

sequence verification. Protein purification was similar to (*37*); briefly, we grew cultures in 50mL of Terrific Broth II with 50mg/mL of kanamycin for 6-12 hours before spiking in IPTG at 1mM final concentration and growing overnight at 18C. Cultures were harvested by spinning at >4000g for 5-10 minutes, and resuspending in lysis buffer (15mL of 25mM Tris-HCl, 300mM NaCl, 40mM Imidazole), with addition of protease inhibitor, lysozyme, and DNAse. Following lysis by sonication and centrifuge at >14,000g for 45 minutes, proteins were purified via nickel Immobilized Metal Affinity Column. Supernatant was allowed to freely drip before washing with 5mL of lysis buffer twice. Proteins were eluted with 1-2mL of elution buffer (25mM Tris-HCl, 300mM NaCl, 500mM Imidazole) before SEC using Superdex 75 10/300GL columns in HBS-EP+ buffer (0.01 M HEPES pH 7.4, 0.15 M NaCl, 3 mM EDTA, 0.005% v/v Surfactant P20, Cytiva #BR100669) and collecting relevant elution fractions.

Surface Plasmon Resonance

Binding kinetics were analyzed via Surface Plasmon Resonance (SPR) on a Biacore 8K (Cytiva). Binding for different pMHC targets was measured by capturing biotinylated peptide-MHC monomer (Fred Hutchinson Cancer Center Immune Monitoring Services) using the Biotin CAPture Kit (Cytiva #28920234). Capture was performed by injecting 0.5 µg/mL pMHC monomer at a flow rate of 10 µL/min in HBS-EP+ (0.01 M HEPES pH 7.4, 0.15 M NaCl, 3 mM EDTA, 0.005% v/v Surfactant P20, Cytiva #BR100669) aiming for a capture level of ~250 response units. Binder analytes in HBS-EP+ buffer were injected at a flow rate of 30 µL/min to monitor association. HBS-EP+ was also used as a running buffer during dissociation under the same flow rate conditions. Ligand concentrations ranged from 1nM to 1uM. Binding kinetics were determined by global fitting of curves to k_{on} and k_{off} assuming a 1:1 Langmuir interaction, using the Cytiva evaluation software.

Chimeric Antigen Receptor constructs

CAR plasmids are constructed based on pSLCAR-CD19-BBZ (Addgene: 135992). In short, the configuration from N-terminal to C-terminal is CD28 signal peptide-FLAG-binder-CD8 hinge-CD28 transmembrane domain-41bb1 cytoplasmic domain-CD3Z cytoplasmic domain-P2A-mTagBFP2-P2A-PuroR.

Mammalian cell culture and transfection

Binder sequences were reverse translated while optimizing for *H. sapiens* and ordered as CAR plasmids from Genscript based on pSLCAR-CD19-BBZ (Addgene: 135992). Lentivirus particles of the CAR plasmids were generated by transfecting 0.25mL HEK 293T cells at 800,000 cell/mL grown in DMEM medium (Gibco, 11965092) supplemented with 10% (v/v) fetal bovine serum (FBS) and 1% (v/v) penicillin–streptomycin (Pen-Strep), with 2 µl TransIT-293 (Mirus), 0.25 µg psPAX2 (Addgene, 12260), 0.1 µg pCMV-VSV-G (Addgene, 8454), and 0.4 µg plasmid in with 50 µl OptiMEM medium (Gibco, 31985070). Transfected HEK293T cells were replenished with fresh RPMI 1640 medium (Gibco, 61870036) supplemented with 10% (v/v) fetal bovine serum

FBS and 1% (v/v) Pen-Strep 24 hours after transfection. Supernatant was collected 48 hours post-transfection and freeze-thawed to kill remaining HEK293T cells. To generate Jurkat-CAR cells, 0.25mL Jurkat cells (a gift from Phil Greenberg lab) at 2 million cells/mL grown in RPMI medium (10% FBS, 1% Pen-Strep) were supplemented with 8 µg/ml Protamine (Millipore Sigma, P4020) and infected with 0.4mL of collected lentivirus supernatant by centrifuging at 1,000g for 60 minutes. Lentivirus-infected Jurkat cells were replenished with fresh RPMI growth medium 24 hours post-infection and every 48 to 72 hours thereafter.

Antigen presenting cell lines
HLA-A*01:01-expressing HEK 293T were made with lentiviral plasmid expressing human HLA-A*01:01 (a gift from Paul Thomas lab); HEK 293T constitutively expressing HLA-A*02:01 were used as HLA-A2 antigen presenting cells. HLA-A*03:01-expressing K-562 cells were made using lentiviral plasmid expression (a gift from Paul Thomas lab).

Yeast surface display clonal binding assay
Clonal yeast surface display samples were obtained by plating sorted pools from FACS on glucose-Trp-Ura agar plates (Teknova C3260), incubating at 30C for 48 hours and picking single colonies. Cultures were grown in C-Trp-Ura (2% glucose w/v) medium and induced for expression in SGCAA (0.2% glucose w/v) medium for 12-18 hours. 50uL of culture was transferred to a 96-well plate and washed with PBSF. Cells were stained at 1:100 v/v with FITC-conjugated anti-C-Myc chicken antibody (ICL, CMYC-45F) for expression and with 1:100 v/v peptide-MHC biotinylated tetramer (Fred Hutchinson Cancer Center Immune Monitoring Services) for both on-target peptide (PE-conjugated) and off-target peptide (APC-conjugated). Cells were incubated for 30 minutes and then washed and resuspended in PBSF. All flow cytometry experiments were performed on the Attune NxT instrument and data was analyzed using the FlowJo software.

Jurkat-CAR binding assay
50 uL of Jurkat-CAR cells at 1 million cells/mL were added to a 96-well plate. Growth medium was removed and replaced with 50 uL staining reagent made of FACS buffer (Dubecco's PBS pH 7.2, 0.5% bovine serum albumin, and 2 mM EDTA) containing 1:100 v/v peptide-MHC biotinylated tetramer (Fred Hutchinson Cancer Center Immune Monitoring Services) for both on-target peptide (PE-conjugated) and off-target peptide (APC-conjugated). Cells were incubated for 30 minutes at 4C and then washed and resuspended in 120uL FACS buffer. All flow cytometry experiments were performed on the Attune NxT instrument and data was analyzed using the FlowJo software. Binding levels were determined by comparing the PE/APC levels of singlet CAR-expressing Jurkat cells (FSC-A/SSC-A gate for cell size, FSC-A/FSC-H gate for singlet, and BFP gate for CAR expressing level)

Activation assays using flow cytometry

Antigen presenting cells (APCs) (HEK293T cells presenting HLA-A*02:01, K652 cells presenting HLA-A*03:01) were resuspended into RPMI medium at 1 million cells/mL and pulsed with peptide to final concentration 10uM (ordered from Genscript and eluted in DMSO) or equivalent volume of DMSO. 100 uL of cells were added to 96-well plates. 100 uL of Jurkat-CAR cells at 1 million cells/mL were added to MHC-presenting cells. Jurkat-CAR cells and MHC-presenting cells were incubated overnight for 12 to 18 hours. After incubation, cells were centrifuged at 800g for 2 minutes to remove supernatant. Cells were stained with 50uL reagent containing anti-mouse CD69 antibody (Biolegend, 104513) conjugated with APC in FACS buffer at 1:100 v/v for 45 minutes in 4C, then washed and resuspended in FACS buffer. CAR-Jurkat activation was measured via flow cytometry (Attune NxT) and data was analyzed using the FlowJo software. Activation levels were determined by comparing the APC levels of singlet CAR-expressing Jurkat cells (FSC-A/SSC-A gate for cell size, FSC-A/FSC-H gate for singlet, and BFP gate for CAR expressing level)

Global peptide scanning

A yeast display HLA-A1 library was generated as previously described (*33*) to display 9-mer peptides, with P3 and P9 as anchoring residues with limited diversity (P3 as aspartate or glutamate, P9 as tyrosine only). For other positions of the peptide library, an NNK codon was used to allow all 20 amino acids. Protein binders were expressed and purified as described with C-terminal 6XHIS tag, then biotinylated. The yeast-display HLA-A1 peptide library was selected with streptavidin-coated magnetic beads coated with biotinylated binder proteins as previously described (*33*).

PBMC Transduction with binder CAR

PBMC were obtained from volunteer donors after written informed consent on Memorial Sloan-Kettering Cancer Center (MSK) Institutional Review Board (IRB) approved protocol 16-312 (approved February 19, 2025). Age, gender, race, and ethnic details are not recorded. PBMCs from HLA-typed healthy donors and patients were obtained by Ficoll density centrifugation. Cells of interest are isolated through negative selection using magnetic microbeads (Miltenyi Biotec). T cells are counted and resuspended in PBS/0.5% FBS/2mM EDTA at 10e7 cells/mL. Cells are incubated with 20uL microbeads per 10e7 cells for 15 minutes at 4oC. LD columns (Miltenyi Biotec #130-042-901) are put in a MACS separator, cells are added to the column and allowed to flow through by gravity. Columns are washed two times with 1mL of PBS/0.5% FBS/2mM EDTA. Cells are spun and resuspended in complete RPMI. T cells that were isolated using negative selection were then stimulated with CD3/CD28 DynabeadsTM (Thermo Fisher Scientific).

10 cm plates were coated with poly-D lysine at room temperature (RT) for 15 minutes and rinsed with sterile water to let dry for 1 hour. 5e6 LentiX (Takara 632180) were plated and incubated overnight to adhere in 10 mL DMEM. 6.7 µg of lentiviral plasmid, 5 µg of psPAX2, and 3.3 µg of

pMD2.G were added to a tube of 750 µL Opti-MEM and 30 µL of P3000 (Invitrogen L3000150) for 5 minutes at RT. In another tube, 750 µL of Opti-MEM and 45 µL of Lipofectamine 3000 (Invitrogen L3000150) were mixed and incubated at RT for 5 minutes. After, both tubes were mixed gently and incubated at RT for 15 minutes. Then, the DMEM was aspirated from the 10 cm plate, and the DNA mixture was added dropwise onto the cells. 10 mL of DMEM was added to the dish afterwards and it was incubated overnight. The next day, the media was aspirated and replaced with 10 mL of fresh RPMI. A 6-well plate was also coated with Retronectin (Takara T100A) overnight in 4C. The following day, the supernatant was collected and replaced with 10 mL of RPMI from the 10cm dish and centrifuged at 500g for 10 minutes. The supernatant was then filtered through a 0.45 µM filter. The Retronectin coated plate was aspirated and blocked with RPMI for 1 hour. Activated CD3+ T cells (Gibco 11131D) were plated on the Retronectin plate at 2e6 per well in 1 mL. 2 mL of filtered virus was added per well with IL-2. Then, cells were centrifuged at 2000g for 1 hour and placed in the incubator overnight. The next day, these steps were repeated, and the cells were incubated with the virus for 3 days. Transduction was checked via flow cytometry for Blue Fluorescent Protein and the cells were ready for downstream applications and the virus was washed away. PBMC were obtained from healthy volunteer donors on MSKCC IRB approved protocol 16-312 (February 19, 2025). Age, gender, race, and ethnic details are not recorded.

Cytotoxicity of pulsed T2 cells.

Target T2 cells expressing firefly luciferase and GFP (fLuc-GFP), were pre-pulsed with peptides at 10ug/ml overnight, washed and were next cocultured with binder-transduced T cells in triplicate at the indicated E:T ratio in the figures using clear bottom, white 96-well assay plates (Corning 3903) with 1 × 104 target cells in a total volume of 200 µl. Target cells alone were plated at the same cell density to determine maximum luciferase activity. Cells were cocultured overnight, at which time d-luciferin substrate (Gold Biotech LUCK) was added at a final concentration of 0.5 µg µl−1 to each well. Emitted light was detected in a Wallac EnVision Multilabel reader (Perkin Elmer). Target lysis was determined as (1 − (RLUsample)/(RLUmax)) × 100 (RLU, relative light unit). Cytotoxicity was measured by bioluminescence emission after adding substrate luciferin at 24 and 48 hours.

Expression, purification and crystallization of pMHC-minibinder complex

Human b2M and HLA-A*02:01 were expressed separately as inclusion bodies in BL21(DE3) cells (Novagen) and went through refolding to form a complex as previously described (*38*, *39*). Briefly, the cells were lysed with ultrasonic sonification, and the inclusion bodies were washed and solubilized in solubilization buffer (8M Urea, 20mM Tris-HCL, pH 8.0, 0.5mM EDTA, 1mM DTT). HLA-A*02:01, b2M and MART1 peptide (Elim; stock prepared in DMSO) were then mixed and added to the refolding buffer (100mM Tris-HCl, pH 8.0, 2mM EDTA, 400mM L-Arginine-HCl, 0.2mM PMSF, 0.5mM oxidized glutathione, 5.0mM reduced glutathione) in molar ratios of 1:2:10. The refolding mixture was incubated overnight at 4°C with stirring, then dialyzed

four times against dialysis buffer (10mM Tris-HCl, pH 8.0). The refolded pMHC mixture was purified using Superdex 200 Increase (GE Healthcare) and MonoQ (GE Healthcare). Finally, Nanobody AD01 and minibinder (treated with carboxypeptidases A and B overnight) were added in excess to the complex, and the mixture was purified again with Superdex 200 Increase before being concentrated to 10mg/ml for crystallization screening.

Rectangular shaped crystals obtained under conditions of 0.2M potassium thiocyanate, 20% w/v PEG 3350 were optimized and harvested for data collection at Stanford Synchrotron Radiation Lightsource beamline 12-1. Datasets were processed by X-ray Detector Software (XDS), and the atomic model was built in COOT and refined with PHENIX software suite.

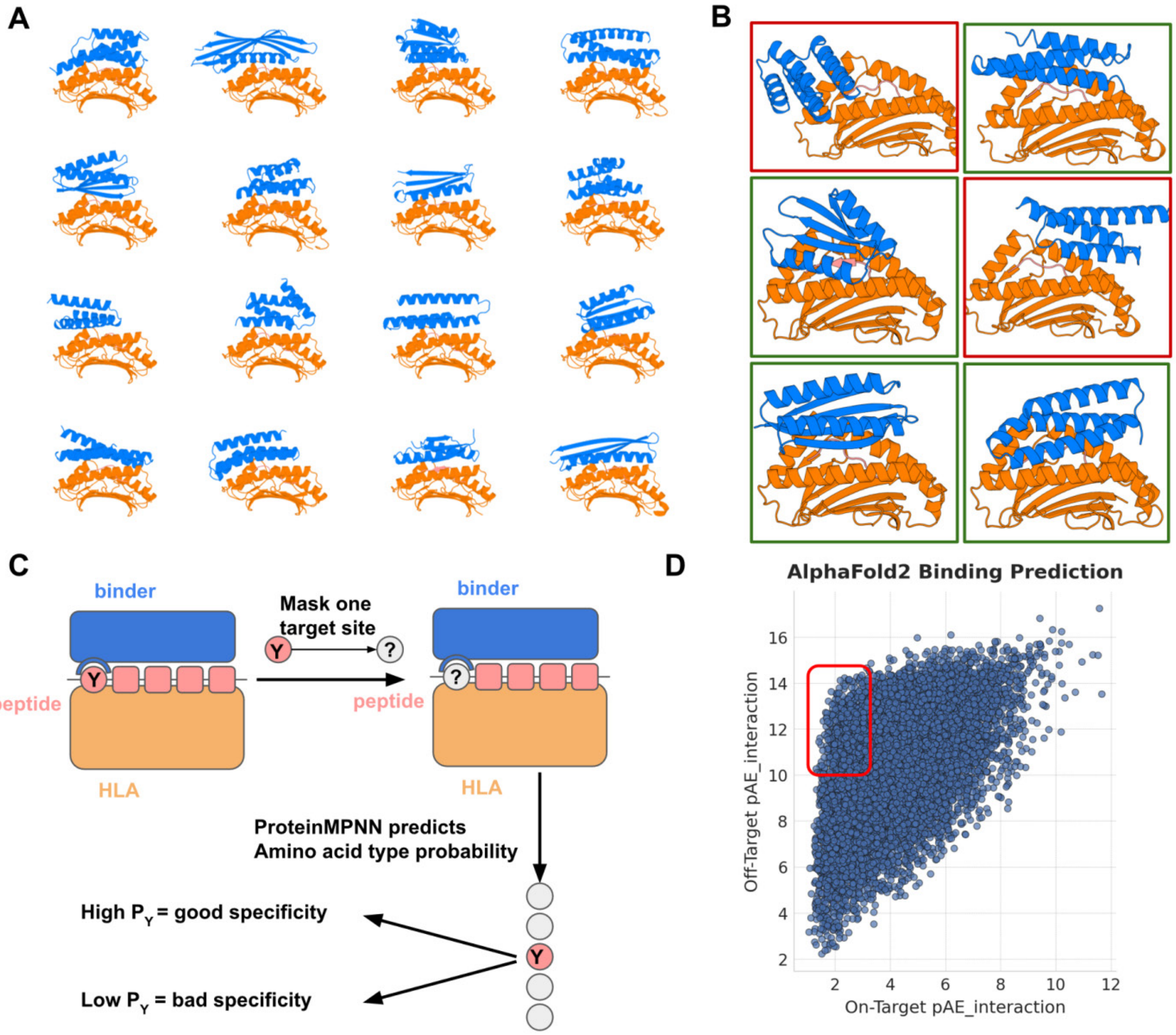
A
B
C
binder
Mask one target site
Y
?
binder
peptide
peptide
HLA
HLA
ProteinMPNN predicts
Amino acid type probability
High P_Y = good specificity
Low P_Y = bad specificity
D
AlphaFold2 Binding Prediction
Off-Target pAE_interaction
On-Target pAE_interaction

Fig. S1. Generation of recyclable scaffold library to peptide-MHC structures. (A) Examples of diverse diffusion scaffolds (blue) on pMHC target (orange). (B) Designs are filtered based on peptide contact area. Designs with extensive peptide contacts (green boxes) are selected, while those with limited peptide contacts (red boxes) are filtered out. (C) Target peptide residue mutation effect evaluation process by ProteinMPNN using predicted binder-pMHC structures. (D) Plot of pAE_interaction values using AF-MHC to fold each design against MHC loaded with target peptides or off-target peptides. Filter (shown as red box) on low interaction pAE_interaction for on-target, high pAE_interaction for off-target.

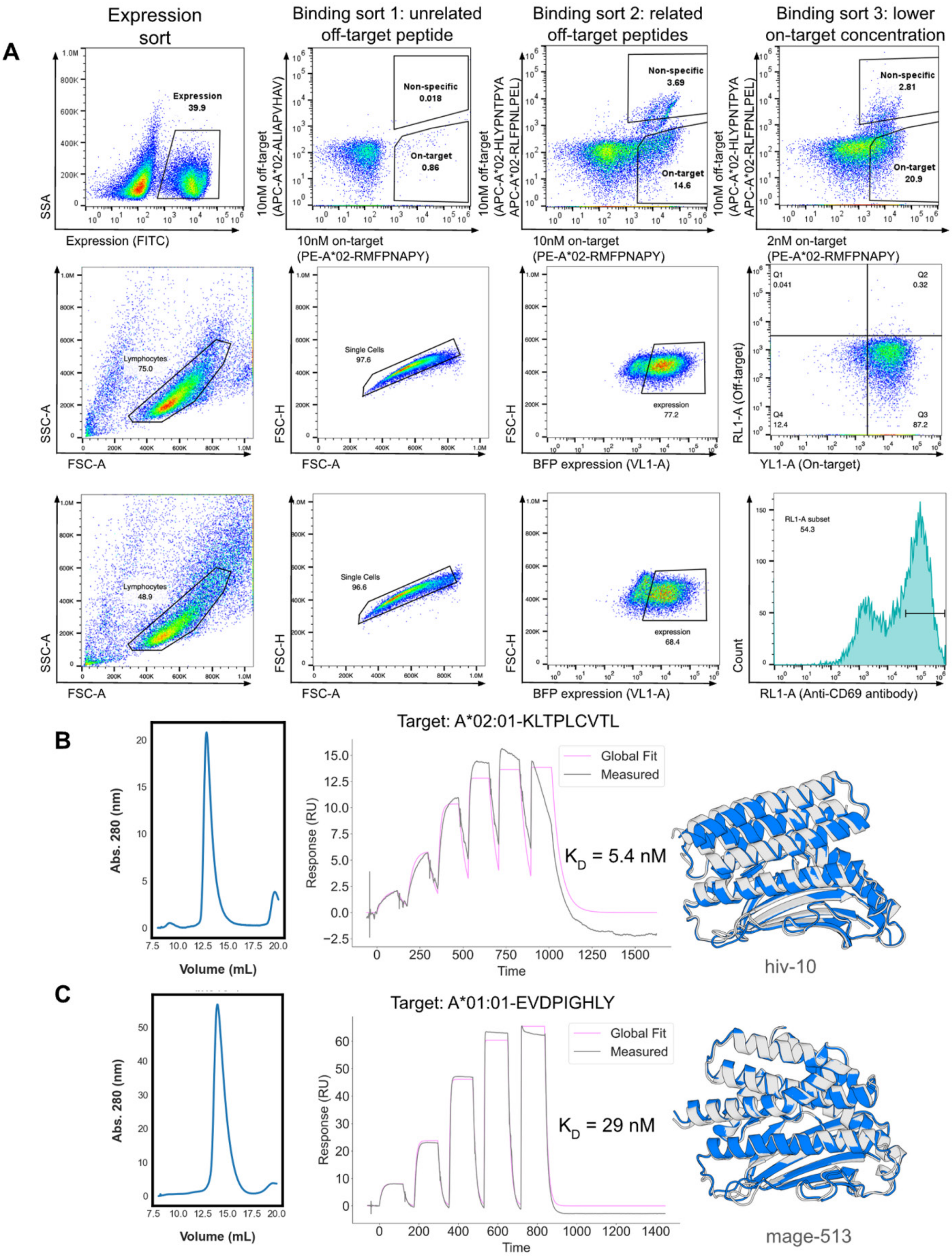
A
Expression sort
Binding sort 1: unrelated off-target peptide
Binding sort 2: related off-target peptides
Binding sort 3: lower on-target concentration
Expression 39.9
SSA
Expression (FITC)
10nM off-target (APC-A*02-ALIAPVHAV)
10nM on-target (PE-A*02-RMFPNAPY)
Non-specific 0.018
On-target 0.86
10nM off-target (APC-A*02-HLYPNTPYA APC-A*02-RLFPNLPEL)
Non-specific 3.69
On-target 14.6
2nM on-target (PE-A*02-RMFPNAPY)
Non-specific 2.81
On-target 20.9
Lymphocytes 75.0
SSC-A
FSC-A
Single Cells 97.6
FSC-H
expression 77.2
BFP expression (VL1-A)
Q1 0.041
Q2 0.32
Q4 12.4
Q3 87.2
RL1-A (Off-target)
YL1-A (On-target)
Lymphocytes 48.9
Single Cells 96.6
expression 68.4
RL1-A subset 54.3
Count
RL1-A (Anti-CD69 antibody)
B
Target: A*02:01-KLTPLCVTL
Abs. 280 (nm)
Volume (mL)
Global Fit
Measured
Response (RU)
Time
K_D = 5.4 nM
hiv-10
C
Target: A*01:01-EVDPIGHLY
K_D = 29 nM
mage-513

Fig. S2. Yeast surface display library screening, Jurkat T cell characterization, and biochemical characterization of individual hits. (A) (Up)WT1 yeast surface display library screening; initial sort for surface expression followed by 3 binding sorts with target pMHC tetramer (x-axis, with decreasing concentrations for later sort) and unrelated or related off-target peptides pMHC tetramers (y-axis). Population in on-target gates was taken for subsequent sorting rounds.(Middle) Example gating strategy for tetramer binding assay. (Bottom) Example gating strategy for CAR activation assay. (B and C) SEC trace, SPR binding kinetics (binding affinity indicated in plot), and design model (blue) overlaid with prediction (gray) for hiv-10 design against A*02:01-KLTPLCVTL (B) and mage-513 design against A*01:01-EVDPIGHLY (C) purified from E. coli expression.

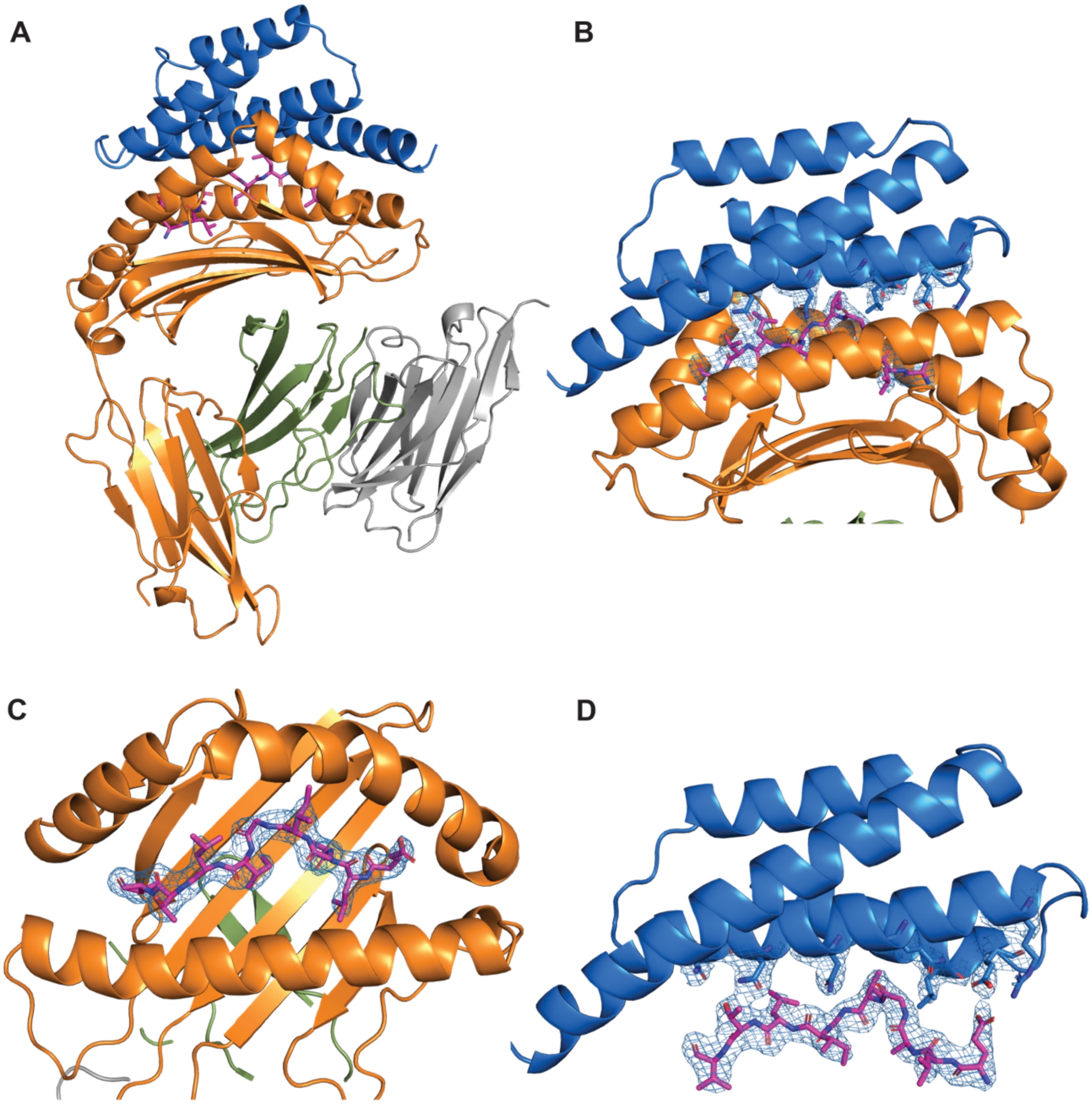
A
B
C
D

Fig. S3. X-ray crystal structure of minibinder mart1-3 in complex with Mart1-HLA:A2 and nanobody AD01. mart1-3 in marine, Mart1 peptide in magenta, HLA:A2 in orange, hB2M in smudge and crystallization chaperon nanobody AD01 in gray. (A) Full complex. (B) Closer view with composite omit map contoured at 1.0 sigma on peptide and interacting residues from minibinder. (C) Top view of peptide in pMHCI grove. (D) mart1-3 with peptide.

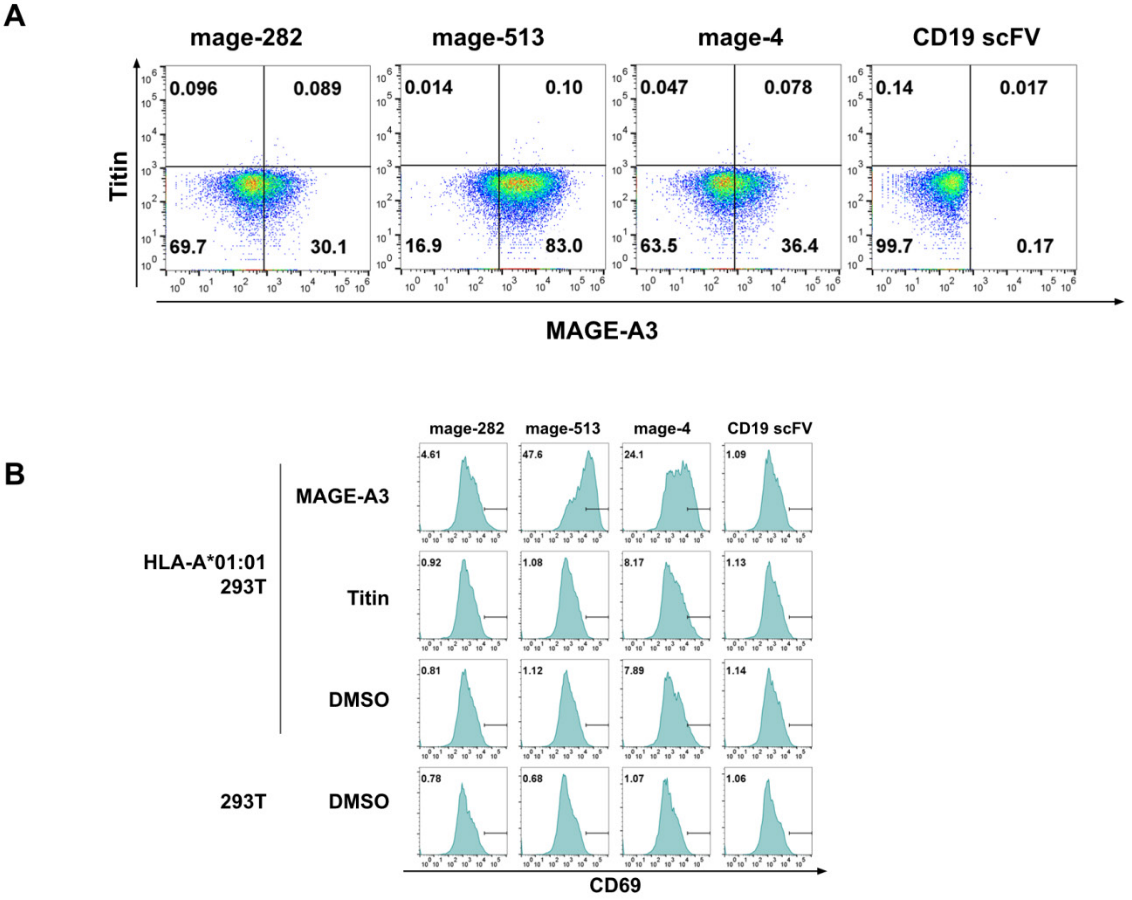
A
mage-282
mage-513
mage-4
CD19 scFV
Titin
MAGE-A3
0.096
0.089
69.7
30.1
0.014
0.10
16.9
83.0
0.047
0.078
63.5
36.4
0.14
0.017
99.7
0.17
B
mage-282
mage-513
mage-4
CD19 scFV
HLA-A*01:01
293T
MAGE-A3
Titin
DMSO
293T
DMSO
4.61
47.6
24.1
1.09
0.92
1.08
8.17
1.13
0.81
1.12
7.89
1.14
0.78
0.68
1.07
1.06
CD69

Fig. S4. Binding and activation of different MAGE-A3 binder based CAR. (A) Flow cytometry of jurkat cells displaying the design expressing 3 MAGE-A3 binders (mage-282, mage-513, mage-4) or CD19 scFV (negative control) based CAR incubated with on-target MAGE-A3 pMHC tetramer (x axis) and off-target Titin tetramers (y axis) at 10nM concentration. (B) Histograms of CD69 level of Jurkat cells expressing 3 MAGE-A3 binders (mage-282, mage-513, mage-4) or CD19 scFV (negative control) based CAR incubated with 293T or HLA-A*01:01 expressing 293T upon pulsing with 5uM indicated peptides (MAGE-A3, Titin) or DMSO. Same flow plots shown in the main figure for mage-513 are included in this figure for comparison purposes

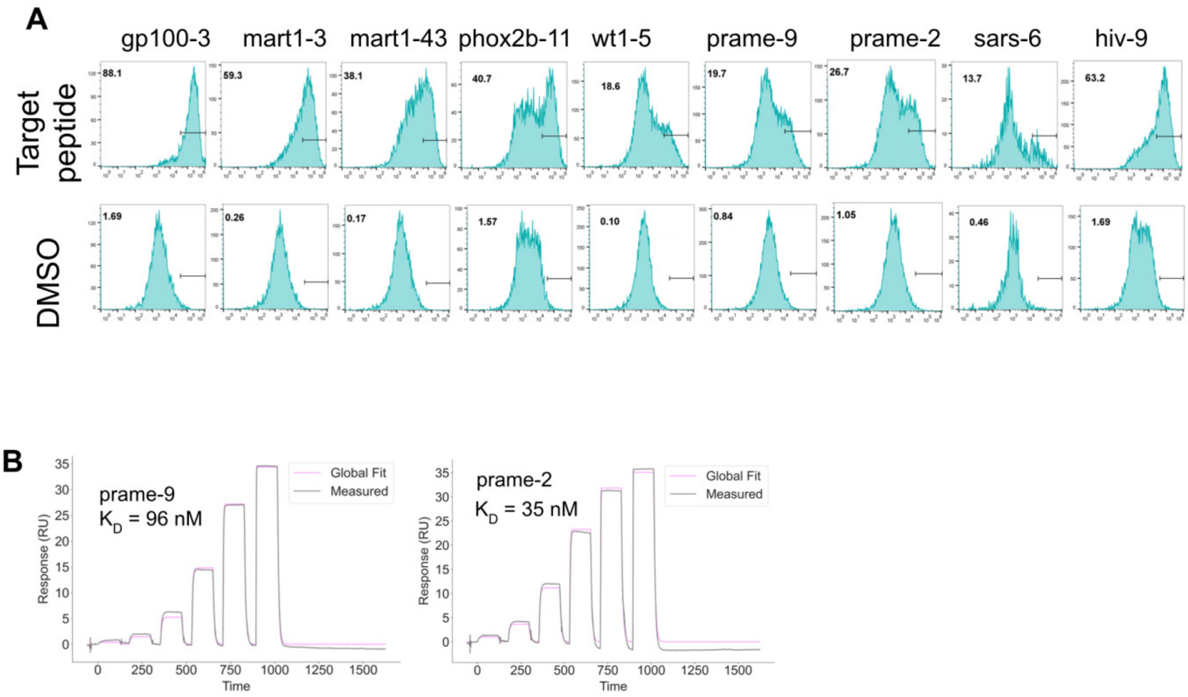
A
gp100-3
mart1-3
mart1-43
phox2b-11
wt1-5
prame-9
prame-2
sars-6
hiv-9
Target peptide
DMSO
88.1
59.3
38.1
40.7
18.6
19.7
26.7
13.7
63.2
1.69
0.26
0.17
1.57
0.10
0.84
1.05
0.46
1.69
B
prame-9
K_D = 96 nM
prame-2
K_D = 35 nM
Global Fit
Measured
Response (RU)
Time

Fig. S5: Biochemical and cellular characterization of the PRAME_A2. (A) CD69 MFI of Jurkat cells expressing indicated CARs (in each column) incubated with 293T upon pulsing with 5uM respective peptide (top) or DMSO (bottom). (B) SPR traces of binder prame-9 and prame-2 on PRAME pMHC monomer.

Allele	Target	On-target peptide	Off-target peptides (genes)
A*01:01	MAGE-A3	EVDPIGHLY	ESDPIVAQY (TTN)
A*02:01	HIV-Env	KLTPLCVTL	KLQELCCTL (YES1)<br>KLNPVCCEL (ATG2B)<br>KLIPLCHQL (CTNNAL1)
A*02:01	Wilms tumor antigen 1	RMFPNAPYL	RLFPNLPEL (ARHGEF11)<br>HLYPNTPYA (APEX1)<br>RMFPTPPSL (MED13L)
A*02:01	SARS-CoV membrane protein	GLMWLSYFV	GLAWLSLFV (KCNK5)<br>GLIFSSYFV (KCNQ1)<br>SLMYLSYLV (ELOVL6)
A*02:01	YFV NS4b214-22	LLWNGPIAV	IVWNGPVGV (PGK2)<br>TLWRGPVVV (STEAP2)
A*02:01	MART-1	ELAGIGILTV	GLMGAGIAQV (HADHA)<br>SLAGLGLWLL (LY6G6C)
A*02:01	gp100	YLEPGPVTA	FQDPVPLTV (TRIM24)<br>YLQPWPVDV (ASB6)
A*02:01	PAP	TLMSAMTNL	TLMSMVANL (PSMB3)<br>TLFSALTGL (ZMPSTE24)
A*02:01	PRAME	ALYVDSLFFL	ILYVDPLPMI(ATP2A1)<br>MLTLNSIFYV (ABCA5)<br>LMYLGSLAFL(ADM)<br>YLSIDSALFV(PQLC1)
A*03:01	CTNNB1 (S5F)	TTAPFLSGK	TTAPSLSGK (CNTTB1)
C*07:02	Phox2B	QYNPIRTTF	QYNPIATTF (PHOX2B)

Table S1.: Peptide-MHC targets and selected off-target peptides. pMHC targets with respective HLA alleles, target peptides, and closely related peptides from the human proteome chosen as off-targets (gene of origin indicated in parentheses).

Binder	Interface_hydrophobic_sasa ($Å^2$)	Interface_polar_sasa ($Å^2$)	mhc_sasa ($Å^2$)	peptide_sasa ($Å^2$)	Design:Prediction RMSD (Å)
yfv-2	2114	892	2692	777	0.82
sars-11	1516	716	1746	756	0.81
hiv-10	1772	934	2359	788	0.93
wt1-8	1545	1044	2248	761	0.72
WT1_6rsy_tcr	1303	1018	1960	769	NA
pap-116	1526	958	2249	580	0.45
ctnnb1-15	1342	579	1729	471	0.46
mage-513	1969	861	2488	793	0.75
MAGE_5brz_tcr	1114	766	1586	581	NA
gp100-3	1739	925	2356	719	0.63
mart1-55	1895	972	2584	826	0.75
Mart1_5nht_tcr	1231	626	1584	764	NA
prame-9	1998	807	2300	880	0.56

Table S2. Design Model Characteristics. Solvent Accessible Surface Area (SASA) values in Å2 for polar residues and hydrophobic residues at the interface for each target/binder pair, compared to TCR values when available. Delta values for SASA of MHC only and Peptide only are shown with MHC minus Peptide. RMSD of the design model to the Chai-1 or AF3 structure.

	mart-1_3-MART1-HLAA2-AD01 complex
**Wavelength (Å)**	0.9795
**Resolution range**	48.12 - 2.27 (2.33 - 2.27)
**Space group**	P 1
**Unit cell (a,b,c (Å)) (α,β,γ(∘))**	87.955 95.863 99.133 89.72 69.93 73.9
**Total reflections**	510361 (38390)
**Unique reflections**	136169 (10407)
**Multiplicity**	3.7 (3.7)
**Completeness (%)**	90.80 (90.53)
**Mean I/sigma(I)**	5.32 (0.38)
**Wilson B-factor**	52.74
**R-merge**	0.1487 (3.289)
**R-meas**	0.1739 (3.874)
**R-pim**	0.08972 (2.03)
**CC1/2**	0.994 (0.648)
**CC***	0.999 (0.887)
**Reflections used in refinement**	122039 (9385)
**Reflections used for R-free**	1796 (146)
**R-work**	0.2069 (0.4162)
**R-free**	0.2342 (0.4531)
**Number of non-hydrogen atoms**	20183
**macromolecules**	19622

**Ligands (glycerol)**	270
**solvent/water molecules**	291
**Protein residues**	2416
**RMS(bonds)**	0.002
**RMS(angles)**	0.5
**Ramachandran favored (%)**	97.77
**Ramachandran outliers (%)**	0.08
**Rotamer outliers (%)**	0.95
**Clashscore**	3.01
**Average B-factor**	82.43
**macromolecules**	82.68
**peptide**	69.94
**minibinder**	104.58
**ligands (glycerol)**	85.23
**solvent/water molecules**	63.1

Table S3. Crystallographic Data collection and refinement statistics for mart-1_3-MART1-HLAA2-AD01 complex.

Data S1. Information of the designed binders. The chart contains binder names, their targets HLA alleles, target peptides, binder amino acid sequences, and if they are coming from the partial diffusion of the same scaffold as mage-513.

Data S2. Next-Generation-Sequencing data of yeast peptide library after four rounds of MAGE-513 selection. The chart contains peptide sequences and raw counts of reads that are responsible for coding the specific peptide sequences.

References and Notes

1. M. Yarmarkovich, Q. F. Marshall, J. M. Warrington, R. Premaratne, A. Farrel, D. Groff, W. Li, M. di Marco, E. Runbeck, H. Truong, J. S. Toor, S. Tripathi, S. Nguyen, H. Shen, T. Noel, N. L. Church, A. Weiner, N. Kendsersky, D. Martinez, R. Weisberg, M. Christie, L. Eisenlohr, K. R. Bosse, D. S. Dimitrov, S. Stevanovic, N. G. Sgourakis, B. R. Kiefel, J. M. Maris, Targeting of intracellular oncoproteins with peptide-centric CARs. *Nature* **623**, 820–827 (2023). doi:10.1038/s41586-023-06706-0 Medline

2. R. Leidner, N. Sanjuan Silva, H. Huang, D. Sprott, C. Zheng, Y.-P. Shih, A. Leung, R. Payne, K. Sutcliffe, J. Cramer, S. A. Rosenberg, B. A. Fox, W. J. Urba, E. Tran, Neoantigen T-cell receptor gene therapy in pancreatic cancer. *N. Engl. J. Med.* **386**, 2112–2119 (2022). doi:10.1056/NEJMoa2119662 Medline

3. E. H.-C. Hsiue, K. M. Wright, J. Douglass, M. S. Hwang, B. J. Mog, A. H. Pearlman, S. Paul, S. R. DiNapoli, M. F. Konig, Q. Wang, A. Schaefer, M. S. Miller, A. D. Skora, P. A. Azurmendi, M. B. Murphy, Q. Liu, E. Watson, Y. Li, D. M. Pardoll, C. Bettegowda, N. Papadopoulos, K. W. Kinzler, B. Vogelstein, S. B. Gabelli, S. Zhou, Targeting a neoantigen derived from a common *TP53* mutation. *Science* **371**, eabc8697 (2021). doi:10.1126/science.abc8697 Medline

4. P. Nathan, J. C. Hassel, P. Rutkowski, J.-F. Baurain, M. O. Butler, M. Schlaak, R. J. Sullivan, S. Ochsenreither, R. Dummer, J. M. Kirkwood, A. M. Joshua, J. J. Sacco, A. N. Shoushtari, M. Orloff, J. M. Piulats, M. Milhem, A. K. S. Salama, B. Curti, L. Demidov, L. Gastaud, C. Mauch, M. Yushak, R. D. Carvajal, O. Hamid, S. E. Abdullah, C. Holland, H. Goodall, S. Piperno-Neumann; IMCgp100-202 Investigators, Overall survival benefit with tebentafusp in metastatic uveal melanoma. *N. Engl. J. Med.* **385**, 1196–1206 (2021). doi:10.1056/NEJMoa2103485 Medline

5. T. Dao, D. Pankov, A. Scott, T. Korontsvit, V. Zakhaleva, Y. Xu, J. Xiang, S. Yan, M. D. de Morais Guerreiro, N. Veomett, L. Dubrovsky, M. Curcio, E. Doubrovina, V. Ponomarev, C. Liu, R. J. O'Reilly, D. A. Scheinberg, Therapeutic bispecific T-cell engager antibody targeting the intracellular oncoprotein WT1. *Nat. Biotechnol.* **33**, 1079–1086 (2015). doi:10.1038/nbt.3349 Medline

6. S. S. Chandran, J. Ma, M. G. Klatt, F. Dündar, C. Bandlamudi, P. Razavi, H. Y. Wen, B. Weigelt, P. Zumbo, S. N. Fu, L. B. Banks, F. Yi, E. Vercher, I. Etxeberria, W. D. Bestman, A. Da Cruz Paula, I. S. Aricescu, A. Drilon, D. Betel, D. A. Scheinberg, B. M. Baker, C. A. Klebanoff, Immunogenicity and therapeutic targeting of a public neoantigen derived from mutated *PIK3CA*. *Nat. Med.* **28**, 946–957 (2022). doi:10.1038/s41591-022-01786-3 Medline

7. M. Glögl, A. Krishnakumar, R. J. Ragotte, I. Goreshnik, B. Coventry, A. K. Bera, A. Kang, E. Joyce, G. Ahn, B. Huang, W. Yang, W. Chen, M. G. Sanchez, B. Koepnick, D. Baker, Target-conditioned diffusion generates potent TNFR superfamily antagonists and agonists. *Science* **386**, 1154–1161 (2024). doi:10.1126/science.adp1779 Medline

8. R. J. Ragotte, M. A. Tortorici, N. J. Catanzaro, A. Addetia, B. Coventry, H. M. Froggatt, J. Lee, C. Stewart, J. T. Brown, I. Goreshnik, J. N. Sims, L. F. Milles, B. I. M. Wicky, M. Glögl, S. Gerben, A. Kang, A. K. Bera, W. Sharkey, A. Schäfer, J. R. Harkema, R. S.

Baric, D. Baker, D. Veesler, Designed miniproteins potently inhibit and protect against MERS-CoV. *Cell Rep.* **44**, 115760 (2025). doi:10.1016/j.celrep.2025.115760 Medline

9. I. Sappington, M. Toul, D. S. Lee, S. A. Robinson, I. Goreshnik, C. McCurdy, T. C. Chan, N. Buchholz, B. Huang, D. Vafeados, N. Roullier, M. Garcia-Sanchez, M. Glögl, C. Kim, J. L. Watson, S. V. Torres, K. H. G. Verschueren, K. Verstraete, C. S. Hinck, M. Bernard-Valle, B. Coventry, J. N. Sims, G. Ahn, X. Wang, A. P. Hinck, T. P. Jenkins, H. Ruohola-Baker, S. Banik, S. N. Savvides, D. Baker, Improved protein binder design using beta-pairing targeted RFdiffusion. bioRxiv 2024.10.11.617496 [Preprint] (2024); https://doi.org/10.1101/2024.10.11.617496.

10. C. Liu, K. Wu, H. Choi, H. Han, X. Zhang, J. L. Watson, S. Shijo, A. K. Bera, A. Kang, E. Brackenbrough, B. Coventry, D. R. Hick, A. N. Hoofnagle, P. Zhu, X. Li, J. Decarreau, S. R. Gerben, W. Yang, X. Wang, M. Lamp, A. Murray, M. Bauer, D. Baker, Diffusing protein binders to intrinsically disordered proteins. bioRxiv 2024.07.16.603789 [Preprint] (2024); https://doi.org/10.1101/2024.07.16.603789.

11. K. Wu, H. Jiang, D. R. Hicks, C. Liu, E. Muratspahic, T. A. Ramelot, Y. Liu, K. McNally, A. Gaur, B. Coventry, W. Chen, A. K. Bera, A. Kang, S. Gerben, M. Y.-L. Lamb, A. Murray, X. Li, M. A. Kennedy, W. Yang, G. Schober, S. M. Brierley, M. H. Gelb, G. T. Montelione, E. Derivery, D. Baker, Design of intrinsically disordered region binding proteins. bioRxiv 2024.07.15.603480 [Preprint] (2024); https://doi.org/10.1101/2024.07.15.603480.

12. L. Cao, B. Coventry, I. Goreshnik, B. Huang, W. Sheffler, J. S. Park, K. M. Jude, I. Marković, R. U. Kadam, K. H. G. Verschueren, K. Verstraete, S. T. R. Walsh, N. Bennett, A. Phal, A. Yang, L. Kozodoy, M. DeWitt, L. Picton, L. Miller, E.-M. Strauch, N. D. DeBouver, A. Pires, A. K. Bera, S. Halabiya, B. Hammerson, W. Yang, S. Bernard, L. Stewart, I. A. Wilson, H. Ruohola-Baker, J. Schlessinger, S. Lee, S. N. Savvides, K. C. Garcia, D. Baker, Design of protein-binding proteins from the target structure alone. *Nature* **605**, 551–560 (2022). doi:10.1038/s41586-022-04654-9 Medline

13. J. Jumper, R. Evans, A. Pritzel, T. Green, M. Figurnov, O. Ronneberger, K. Tunyasuvunakool, R. Bates, A. Žídek, A. Potapenko, A. Bridgland, C. Meyer, S. A. A. Kohl, A. J. Ballard, A. Cowie, B. Romera-Paredes, S. Nikolov, R. Jain, J. Adler, T. Back, S. Petersen, D. Reiman, E. Clancy, M. Zielinski, M. Steinegger, M. Pacholska, T. Berghammer, S. Bodenstein, D. Silver, O. Vinyals, A. W. Senior, K. Kavukcuoglu, P. Kohli, D. Hassabis, Highly accurate protein structure prediction with AlphaFold. *Nature* **596**, 583–589 (2021). doi:10.1038/s41586-021-03819-2 Medline

14. A. Motmaen, J. Dauparas, M. Baek, M. H. Abedi, D. Baker, P. Bradley, Peptide-binding specificity prediction using fine-tuned protein structure prediction networks. *Proc. Natl. Acad. Sci. U.S.A.* **120**, e2216697120 (2023). doi:10.1073/pnas.2216697120 Medline

15. Chai Discovery, J. Boitreaud, J. Dent, M. McPartlon, J. Meier, V. Reis, A. Rogozhnikov, K. Wu, Chai-1: Decoding the molecular interactions of life. bioRxiv 2024.10.10.615955 [Preprint] (2024); https://doi.org/10.1101/2024.10.10.615955.

16. J. Abramson, J. Adler, J. Dunger, R. Evans, T. Green, A. Pritzel, O. Ronneberger, L. Willmore, A. J. Ballard, J. Bambrick, S. W. Bodenstein, D. A. Evans, C.-C. Hung, M. O'Neill, D. Reiman, K. Tunyasuvunakool, Z. Wu, A. Žemgulytė, E. Arvaniti, C. Beattie,

O. Bertolli, A. Bridgland, A. Cherepanov, M. Congreve, A. I. Cowen-Rivers, A. Cowie, M. Figurnov, F. B. Fuchs, H. Gladman, R. Jain, Y. A. Khan, C. M. R. Low, K. Perlin, A. Potapenko, P. Savy, S. Singh, A. Stecula, A. Thillaisundaram, C. Tong, S. Yakneen, E. D. Zhong, M. Zielinski, A. Žídek, V. Bapst, P. Kohli, M. Jaderberg, D. Hassabis, J. M. Jumper, Accurate structure prediction of biomolecular interactions with AlphaFold 3. *Nature* **630**, 493–500 (2024). doi:10.1038/s41586-024-07487-w Medline

17. J. Liu, Y. Sun, J. Qi, F. Chu, H. Wu, F. Gao, T. Li, J. Yan, G. F. Gao, The membrane protein of severe acute respiratory syndrome coronavirus acts as a dominant immunogen revealed by a clustering region of novel functionally and structurally defined cytotoxic T-lymphocyte epitopes. *J. Infect. Dis.* **202**, 1171–1180 (2010). doi:10.1086/656315 Medline

18. A. Bovay, V. Zoete, P. J. Rizkallah, K. Beck, P. Delbreil, D. E. Speiser, D. K. Cole, S. A. Fuertes Marraco, Identification of a superagonist variant of the immunodominant Yellow fever virus epitope $NS4b_{214-222}$ by combinatorial peptide library screening. *Mol. Immunol.* **125**, 43–50 (2020). doi:10.1016/j.molimm.2020.06.025 Medline

19. P. H. N. Celie, M. Toebes, B. Rodenko, H. Ovaa, A. Perrakis, T. N. M. Schumacher, UV-induced ligand exchange in MHC class I protein crystals. *J. Am. Chem. Soc.* **131**, 12298–12304 (2009). doi:10.1021/ja9037559 Medline

20. D. Bloemberg, T. Nguyen, S. MacLean, A. Zafer, C. Gadoury, K. Gurnani, A. Chattopadhyay, J. Ash, J. Lippens, D. Harcus, M. Pagé, A. Fortin, R. A. Pon, R. Gilbert, A. Marcil, R. D. Weeratna, S. McComb, A high-throughput method for characterizing novel chimeric antigen receptors in Jurkat cells. *Mol. Ther. Methods Clin. Dev.* **16**, 238–254 (2020). doi:10.1016/j.omtm.2020.01.012 Medline

21. C. J. Holland, R. M. Crean, J. M. Pentier, B. de Wet, A. Lloyd, V. Srikannathasan, N. Lissin, K. A. Lloyd, T. H. Blicher, P. J. Conroy, M. Hock, R. J. Pengelly, T. E. Spinner, B. Cameron, E. A. Potter, A. Jeyanthan, P. E. Molloy, M. Sami, M. Aleksic, N. Liddy, R. A. Robinson, S. Harper, M. Lepore, C. R. Pudney, M. W. van der Kamp, P. J. Rizkallah, B. K. Jakobsen, A. Vuidepot, D. K. Cole, Specificity of bispecific T cell receptors and antibodies targeting peptide-HLA. *J. Clin. Invest.* **130**, 2673–2688 (2020). doi:10.1172/JCI130562 Medline

22. L. Gao, I. Bellantuono, A. Elsässer, S. B. Marley, M. Y. Gordon, J. M. Goldman, H. J. Stauss, Selective elimination of leukemic $CD34^+$ progenitor cells by cytotoxic T lymphocytes specific for WT1. *Blood* **95**, 2198–2203 (2000). doi:10.1182/blood.V95.7.2198 Medline

23. T. Dao, S. Yan, N. Veomett, D. Pankov, L. Zhou, T. Korontsvit, A. Scott, J. Whitten, P. Maslak, E. Casey, T. Tan, H. Liu, V. Zakhaleva, M. Curcio, E. Doubrovina, R. J. O'Reilly, C. Liu, D. A. Scheinberg, Targeting the intracellular WT1 oncogene product with a therapeutic human antibody. *Sci. Transl. Med.* **5**, 176ra33 (2013). doi:10.1126/scitranslmed.3005661 Medline

24. B. M. Olson, T. P. Frye, L. E. Johnson, L. Fong, K. L. Knutson, M. L. Disis, D. G. McNeel, HLA-A2-restricted T-cell epitopes specific for prostatic acid phosphatase. *Cancer Immunol. Immunother.* **59**, 943–953 (2010). doi:10.1007/s00262-010-0820-6 Medline

25. M. S. Miller, J. Douglass, M. S. Hwang, A. D. Skora, M. Murphy, N. Papadopoulos, K. W. Kinzler, B. Vogelstein, S. Zhou, S. B. Gabelli, An engineered antibody fragment

targeting mutant β-catenin via major histocompatibility complex I neoantigen presentation. *J. Biol. Chem.* **294**, 19322–19334 (2019). doi:10.1074/jbc.RA119.010251 Medline

26. S. Vázquez Torres, P. J. Y. Leung, P. Venkatesh, I. D. Lutz, F. Hink, H.-H. Huynh, J. Becker, A. H.-W. Yeh, D. Juergens, N. R. Bennett, A. N. Hoofnagle, E. Huang, M. J. MacCoss, M. Expòsit, G. R. Lee, A. K. Bera, A. Kang, J. De La Cruz, P. M. Levine, X. Li, M. Lamb, S. R. Gerben, A. Murray, P. Heine, E. N. Korkmaz, J. Nivala, L. Stewart, J. L. Watson, J. M. Rogers, D. Baker, De novo design of high-affinity binders of bioactive helical peptides. *Nature* **626**, 435–442 (2024). doi:10.1038/s41586-023-06953-1 Medline

27. B. Gaugler, B. Van den Eynde, P. van der Bruggen, P. Romero, J. J. Gaforio, E. De Plaen, B. Lethé, F. Brasseur, T. Boon, Human gene MAGE-3 codes for an antigen recognized on a melanoma by autologous cytolytic T lymphocytes. *J. Exp. Med.* **179**, 921–930 (1994). doi:10.1084/jem.179.3.921 Medline

28. M. C. C. Raman, P. J. Rizkallah, R. Simmons, Z. Donnellan, J. Dukes, G. Bossi, G. S. Le Provost, P. Todorov, E. Baston, E. Hickman, T. Mahon, N. Hassan, A. Vuidepot, M. Sami, D. K. Cole, B. K. Jakobsen, Direct molecular mimicry enables off-target cardiovascular toxicity by an enhanced affinity TCR designed for cancer immunotherapy. *Sci. Rep.* **6**, 18851 (2016). doi:10.1038/srep18851 Medline

29. B. J. Cameron, A. B. Gerry, J. Dukes, J. V. Harper, V. Kannan, F. C. Bianchi, F. Grand, J. E. Brewer, M. Gupta, G. Plesa, G. Bossi, A. Vuidepot, A. S. Powlesland, A. Legg, K. J. Adams, A. D. Bennett, N. J. Pumphrey, D. D. Williams, G. Binder-Scholl, I. Kulikovskaya, B. L. Levine, J. L. Riley, A. Varela-Rohena, E. A. Stadtmauer, A. P. Rapoport, G. P. Linette, C. H. June, N. J. Hassan, M. Kalos, B. K. Jakobsen, Identification of a Titin-derived HLA-A1–presented peptide as a cross-reactive target for engineered MAGE A3–directed T cells. *Sci. Transl. Med.* **5**, 197ra103 (2013). doi:10.1126/scitranslmed.3006034 Medline

30. A. B. Bakker, M. W. Schreurs, G. Tafazzul, A. J. de Boer, Y. Kawakami, G. J. Adema, C. G. Figdor, Identification of a novel peptide derived from the melanocyte-specific gp100 antigen as the dominant epitope recognized by an HLA-A2.1-restricted anti-melanoma CTL line. *Int. J. Cancer* **62**, 97–102 (1995). doi:10.1002/ijc.2910620118 Medline

31. Y. Kawakami, S. Eliyahu, K. Sakaguchi, P. F. Robbins, L. Rivoltini, J. R. Yannelli, E. Appella, S. A. Rosenberg, Identification of the immunodominant peptides of the MART-1 human melanoma antigen recognized by the majority of HLA-A2-restricted tumor infiltrating lymphocytes. *J. Exp. Med.* **180**, 347–352 (1994). doi:10.1084/jem.180.1.347 Medline

32. K. Rezvani, A. S. M. Yong, A. Tawab, B. Jafarpour, R. Eniafe, S. Mielke, B. N. Savani, K. Keyvanfar, Y. Li, R. Kurlander, A. J. Barrett, Ex vivo characterization of polyclonal memory $CD8^+$ T-cell responses to PRAME-specific peptides in patients with acute lymphoblastic leukemia and acute and chronic myeloid leukemia. *Blood* **113**, 2245–2255 (2009). doi:10.1182/blood-2008-03-144071 Medline

33. M. E. Birnbaum, J. L. Mendoza, D. K. Sethi, S. Dong, J. Glanville, J. Dobbins, E. Ozkan, M. M. Davis, K. W. Wucherpfennig, K. C. Garcia, Deconstructing the peptide-MHC

specificity of T cell recognition. *Cell* **157**, 1073–1087 (2014). doi:10.1016/j.cell.2014.03.047 Medline

34. M. H. Gee, A. Han, S. M. Lofgren, J. F. Beausang, J. L. Mendoza, M. E. Birnbaum, M. T. Bethune, S. Fischer, X. Yang, R. Gomez-Eerland, D. B. Bingham, L. V. Sibener, R. A. Fernandes, A. Velasco, D. Baltimore, T. N. Schumacher, P. Khatri, S. R. Quake, M. M. Davis, K. C. Garcia, Antigen identification for orphan T cell receptors expressed on tumor-infiltrating lymphocytes. *Cell* **172**, 549–563.e16 (2018). doi:10.1016/j.cell.2017.11.043 Medline

35. 77forest, 77forest/pmhci_binder_design: Pmhci_binder_design, v1.0.0, Zenodo (2025); https://doi.org/10.5281/zenodo.15169815.

36. B. Reynisson, B. Alvarez, S. Paul, B. Peters, M. Nielsen, NetMHCpan-4.1 and NetMHCIIpan-4.0: Improved predictions of MHC antigen presentation by concurrent motif deconvolution and integration of MS MHC eluted ligand data. *Nucleic Acids Res.* **48**, W449–W454 (2020). doi:10.1093/nar/gkaa379 Medline

37. B. I. M. Wicky, L. F. Milles, A. Courbet, R. J. Ragotte, J. Dauparas, E. Kinfu, S. Tipps, R. D. Kibler, M. Baek, F. DiMaio, X. Li, L. Carter, A. Kang, H. Nguyen, A. K. Bera, D. Baker, Hallucinating symmetric protein assemblies. *Science* **378**, 56–61 (2022). doi:10.1126/science.add1964 Medline

38. D. N. Garboczi, D. T. Hung, D. C. Wiley, HLA-A2–peptide complexes: Refolding and crystallization of molecules expressed in *Escherichia coli* and complexed with single antigenic peptides. *Proc. Natl. Acad. Sci. U.S.A.* **89**, 3429–3433 (1992). doi:10.1073/pnas.89.8.3429 Medline

39. X. Yang, D. Nishimiya, S. Löchte, K. M. Jude, M. Borowska, C. S. Savvides, M. Dougan, L. Su, X. Zhao, J. Piehler, K. C. Garcia, Facile repurposing of peptide–MHC-restricted antibodies for cancer immunotherapy. *Nat. Biotechnol.* **41**, 932–943 (2023). doi:10.1038/s41587-022-01567-w Medline
